# Water Availability and Hydraulic Strategies Control Leaf Thermoregulation and Damage During Heat Stress and Recovery in Temperate Tree Species

**DOI:** 10.1111/pce.70605

**Published:** 2026-05-17

**Authors:** Jana K. Zeppan, Lisa Hülsmann, Timo Knüver, Yanick Ziegler, Nadine K. Ruehr

**Affiliations:** ^1^ Institute of Meteorology and Climate Research ‐ Atmospheric Environmental Research (IMK‐IFU), KIT‐Campus Alpin Karlsruhe Institute of Technology (KIT) Garmisch‐Partenkirchen Germany; ^2^ Ecosystem Analysis and Simulation (EASI) Lab University of Bayreuth Bayreuth Germany; ^3^ Bayreuth Centre of Ecology and Environmental Research (BayCEER) University of Bayreuth Bayreuth Germany; ^4^ Institute of Geography and Geoecology (IfGG) Karlsruhe Institute of Technology (KIT) Karlsruhe Germany

**Keywords:** drought, gas exchange, heat, transpirational cooling

## Abstract

Heatwaves are intensifying worldwide, often coinciding with high vapour pressure deficit (VPD) and soil drought. Yet, how temperate tree species tolerate these combined stresses remains elusive. Using single‐tree gas‐exchange chambers, we examined the response of leaf gas exchange and thermoregulation of three broadleaved tree species to a stepwise increase in air temperature (25°C–45°C) along with VPD (2.5–7.9 kPa) under well‐watered and moderate drought conditions, followed by a 2‐day recovery at 25°C. Across species, heat stress increased transpiration but reduced net assimilation, leading to sharp declines in water‐use efficiency. Alongside, leaf cooling intensified, driving leaf temperatures below air temperature. Under moderate drought, restricted stomatal conductance reduced transpirational cooling, causing leaf temperatures to exceed air temperature and increasing heat‐related damage. Species differed markedly in their heat responses along the isohydric–anisohydric spectrum. Isohydric *Acer platanoides* showed early stomatal closure, limited cooling, severe leaf damage, and poor recovery. Anisohydric *Quercus robur* maintained gas exchange, exhibited minimal leaf damage and recovered rapidly, while *Fagus sylvatica* showed intermediate responses. Together, these results highlight that the ability of temperate trees to withstand future heatwaves will depend on the interplay between water availability, transpirational cooling, as well as species‐specific hydraulic strategy and heat tolerance.

## Summary statement

Water availability modulates heat stress responses in three temperate tree species with drought constraining gas exchange and leaf cooling, and increasing damage. Species differences along the isohydric–anisohydric spectrum further determine heat tolerance and recovery.

## 1 Introduction

The past decade has seen the hottest and driest years on record, with extreme summer heatwaves projected to further increase in magnitude and frequency, particularly in mid‐latitude regions (Domeisen et al. 2022; Knutzen et al. 2025). Heat waves not only elevate air temperatures but also drive increases in vapour pressure deficit (VPD) and intensify atmospheric water demand. Moreover, they are frequently preceded and accompanied by soil drought. Compound heat and drought events impose severe constraints on both carbon and water fluxes in forests, leading to widespread tree damage (Ameye et al. 2012; Marchin et al. 2022; Ruehr et al. 2016). In response to these compound stressor, common temperate tree species in Europe such as *Picea abies H. Karst* or *Fagus sylvatica L*. have exhibited significant crown damage in recent years (BMEL 2024; ICP forests 2024). Consequently, there is growing interest in identifying alternative tree species that can withstand climate extremes (Franceschi et al. 2023; Fuchs et al. 2021; Kunz et al. 2016). However, while much research has focused on drought stress and its physiological consequences on a variety of tree species, impacts of heat stress itself and heat‐drought interactions remain underexplored (Breshears et al. 2021).

All physiological processes from cellular to whole‐plant level are strongly temperature‐dependent (Jagadish et al. 2021) and increasing growing temperatures are modifying growth, gas exchange and water relations (Rubio et al. 2025). As air temperatures (T_air_) rise, leaf temperatures (T_leaf_) follow, and the fine‐tuned coupling of carbon assimilation and water loss is increasingly disrupted (Griebel et al. 2020). Commonly, net assimilation (A_net_) and transpiration (*E*) are closely coupled at the leaf‐level via stomatal conductance (g_s_) (Farquhar and Sharkey 1982). However, increasing evidence indicates that during heatwaves *E* continues or even increases while A_net_ declines, leading to a decrease in apparent water‐use efficiency (WUE_a_; A_net_/*E*)(Marchin et al. 2023; Urban et al. 2017). Whether this sustained *E* is an active response driven by increased stomatal opening at high temperatures or a passive consequence of elevated VPD and consequent rise in atmospheric water demand and fluidity remains a topic of debate (Diao et al. 2024; Grossiord et al. 2020; Marchin et al. 2023). To better interpret changes in WUE_a_, intrinsic water use efficiency (WUE_i_ = A_net_/g_s_) can be assessed alongside, as WUE_i_ helps distinguish shifts driven by stomatal regulation from those caused by non‐stomatal constraints on photosynthesis. Irrespective of the underlying mechanism, the decoupling between A_net_ and *E* presents a physiological trade‐off: while excessive water loss can accelerate hydraulic damages, sustained *E* facilitates transpirational cooling and mitigates the risk of thermal damage during heatwaves (Cook et al. 2021; Guha et al. 2018). Thereby, trees can prevent excessive leaf heating and keep T_leaf_ closer to the photosynthetic optimum, while reducing the risk of leaf scorching (Drake et al. 2018; Fauset et al. 2018). Even though the role of *E* in leaf temperature regulation is well‐established, few studies have directly quantified its impact on T_leaf_ and how the relationship between A_net_, *E* and g_s_ changes with increasing heat stress in multiple species.

A tree's ability to tolerate heat stress is strongly determined by water availability (Bauweraerts et al. 2014; Cook et al. 2021; Ruehr et al. 2016). Under drought conditions, stomatal closure limits *E* to preserve water status, reduce hydraulic risk and maintain turgor for metabolic and growth functions (Martin‐StPaul et al. 2017; Peters et al. 2023). However, this strategy also reduces A_net_ and compromises leaf cooling, often leading to T_leaf_ well above T_air_. As a result, trees under combined drought and heat stress experience higher T_leaf_, increased stomatal and photochemical limitations to carbon uptake and a greater risk of heat‐induced leaf damage compared to heat stress alone (Al‐Salman et al. 2023; Marchin et al. 2022; Ruehr et al. 2016). To maintain carbon assimilation, moderately drought‐stressed trees typically exhibit increased WUE to optimise carbon uptake relative to water loss. However, extreme heat disrupts this strategy by increasing transpirational demands while simultaneously suppressing A_net_ after the optimum temperature for photosynthesis (T_opt_) is reached, leading to a steep decline in WUE (Birami et al. 2020). Recent findings suggest that this is even the case under severe drought, where g_s_ remains elevated even when A_net_ approaches zero (Gauthey et al. 2024; Marchin et al. 2023), further challenging the expected relationship between A_net_, g_s_ and *E*. A clearer understanding of these gas exchange dynamics is essential, as it remains uncertain how drought‐induced stomatal responses interact with rising temperatures to shape leaf cooling capacity, net assimilation, and heat‐induced leaf damage.

Apart from soil water availability, interspecific differences in stomatal regulation and heat tolerance influence the response to heat and drought stress. These can be categorised along the isohydric‐anisohydric continuum, reflecting the degree to which species prioritise water conservation versus stomatal opening and therefore leaf cooling (Guo et al. 2025). Species at the isohydric end of the spectrum are characterised by tight stomatal closing under soil or atmospheric drought. They are typically more conservative in their water‐use but this may come at the cost of reduced leaf cooling. In contrast, anisohydric species keep stomata open during drought and may thus be more effective at leaf cooling (Meinzer et al. 2016; Tardieu and Simonneau 1998). While isohydric behaviour maintains relatively stable leaf water potentials through early stomatal closure, it does not necessarily confer greater drought tolerance. Instead, anisohydric species commonly show stronger drought resistance (Chen et al. 2022) and recent findings on desert shrubs indicate that anisohydric species also exhibit greater thermal tolerance of the leaves (Guo et al. 2025). This might, among others, be linked to distinct diurnal patterns related to the species‐specific hydraulic strategy, for example greater morning stomatal opening at low VPD followed by reduced conductance at midday (Matthews et al. 2017). Addressing these underlying mechanisms is critical to anticipate the future of European temperate tree species with distinct strategies under warmer and drier conditions.

Beyond the immediate stress response, a tree's ability to recover its physiological functioning following heat stress is critical to avoid cumulative damage, particularly as extreme events become more frequent (Ruehr et al. 2016; Schwalm et al. 2017). Recovery rates can vary significantly depending on the intensity of the stress. For instance, combined heat and drought tends to slow down recovery rates compared to single stress, due to compound hydraulic and metabolic constraints (Birami et al. 2018; Rehschuh and Ruehr 2022). Simultaneously, trees experiencing lower damage during heat waves are more likely to restore gas exchange quickly (Ruehr et al. 2019). Further, species‐specific differences play a role, with ring‐porous trees showing a faster recovery in their growth compared to diffuse‐porous species (Kannenberg et al. 2019). Still, our understanding of how leaf level gas exchange recovers from heat stress across tree species with contrasting hydraulic strategies remains incomplete.

Therefore, we still lack integrated assessments of leaf thermoregulation and gas exchange during and after heat stress in combination with limited water availability, particularly across species along the isohydric‐anisohydric spectrum. To address these knowledge gaps, we conducted a controlled single‐tree chamber experiment examining the heat and hot drought response of seedlings of three temperate broadleaved tree species native to Europe: *Acer platanoides L., Fagus sylvatica L*. and *Quercus robur L*. along the isohydric‐anisohydric continuum. More specifically, we investigated seedling responses with respect to continuous leaf gas exchange, leaf temperature and leaf damage. Seedlings were exposed to a step‐wise increase in temperature (25°C–45°C) and VPD under either well‐watered or moderate drought conditions. We hypothesised that:

1. Heat increases transpirational cooling while reducing WUE when water supply is abundant, but under combined heat‐drought reduced stomatal conductance limits cooling and leads to stronger leaf damage.
2. Anisohydric species (*Q. robur* and *F. sylvatica*) tolerate higher temperatures by sustaining carbon assimilation and leaf cooling longer than isohydric *A. platanoides*.
3. Post‐heat recovery of leaf gas exchange is constrained by soil water availability and the extent of heat‐induced leaf damage.

## 2 Methods

### 2.1 Plant Material and Growth Conditions

Seeds of *A. platanoides*, *Q. robur* and *F. sylvatica* (Provenience 80004, 81709 and 81024, respectively) were sown in February 2022 in separate trays filled with a carbon‐free mixture of fine (3–4 mm) and coarse vermiculite (7‐8 mm) in a 1:2 ratio. Once seedlings had germinated, they were transferred to small pots (0.5 L) with fine and coarse vermiculite in a 1:1 ratio. Substrate was supplemented by 5 g of slow‐release fertiliser (Osmocote Exact 3–4 M 16‐9‐12 + 2MgO + TE; ICL Specialty Fertilisers, Heerlen, the Netherland). Throughout the experiment, seedlings were grown in carbon‐free potting substrate to minimise CO_2_ background signals from the substrate to avoid heterotrophic respiration signals from decomposition of soil organic carbon, thereby facilitating root respiration measurements within the belowground compartments of the Tree Flux system. In May 2022, seedlings were transplanted to 3.5 L pots containing a substrate mixture of coarse vermiculite (7–8 mm), fine vermiculite (2–3 mm), medium‐grained quartz sand (1–2 mm) and coarse gravel (4–6 mm) in a 3:1:1:1 ratio. Due to its sensitivity for water logging, substrate mixture for *F. sylvatica* was slightly adjusted to a mixing ratio of 2:1:2:1 of coarse vermiculite, perlite, medium‐grained quartz sand and coarse gravel. Substrate was supplemented by 10 g tree^‐1^ of the same slow‐release fertiliser.

Throughout the germination and growth phase, seedlings were kept within the greenhouse facilities of the KIT‐Campus Alpin in Garmisch‐Partenkirchen, Germany (732 m asl, 47°28032.87"N, 11°3044.03"E). Mean daytime (10:00−18:00) temperature during the growth period from February to June was 20°C, photosynthetic active radiation (PAR) was 260 µmol m^‐2^ m^‐1^, and relative humidity was 75%. During the cultivation phase, the seedlings were watered to field capacity regularly using an automated drip‐irrigation system to ensure equal watering of all individuals.

### 2.2 Experimental Design

Species were subjected to the heat stress experiment in separate runs due to the limited number of available gas exchange chambers (*n* = 20). First, seedlings of *A. platanoides*, then *Q. robur*, and lastly *F. sylvatica* were transferred into the chambers of the Tree Flux System (see below) starting in July 2022. Two weeks before starting the heat experiment for each species, seedlings were assigned to two watering treatments: a well‐watered and a moderate drought treatment. Due to technical constraints, irrigation could only be applied at the treatment level, rather than per individual tree. Because seedlings differed in size, individuals with greater leaf biomass were assigned to the well‐watered treatment (Supporting Information Table S1) in order to maintain the targeted soil water content. To verify that this allocation did not introduce physiological bias, we measured leaf‐level gas exchange prior to treatment imitation and detected no differences among treatments.

Watering was performed based on the relative soil water content (RSWC):

$$\mathrm{RSWC}\;=\;100\;\cdot \;\frac{{\mathrm{SWC}}_{\mathrm{sample}}}{{\mathrm{SWC}}_{\max}},$$

where *SWC*_sample_ is the amount of H_2_O (g L^−1^) in the substrate of the given sample and *SWC*_max_ the maximum amount of water (g L^‐1^) the substrate can hold.

Plants in the well‐watered treatment were watered to 80% RSWC while watering of the drought treatment was restricted to 40% RSWC. As soil mixture differed for *F. sylvatica* and preliminary observations indicated that high RSWC can cause root rotting in beech seedlings, RSWC was set to 60% for well‐watered and 30% for drought trees, thereby maintaining the RSWC ratio of 2:1 between the well‐watered and drought treatment within each species. To keep the SWC constant, seedlings were watered three times per day (12am, 8am, 4 pm) using an automated drip irrigation system. The amount of irrigation was adapted within each treatment to maintain the targeted SWC, resulting in an increased irrigation volume at higher temperature, to compensate for increased transpiration demand and associated soil water depletion. Due to differences in germination success, the number of seedlings for each watering treatment and species varied: *A. platanoides* (well‐watered: *n* = 7 and drought: *n* = 6), *Q. robur* (well‐watered: *n* = 7 and drought *n* = 7), and *F. sylvatica* (well‐watered: *n* = 10 and drought: *n* = 9).

**Figure 1:**
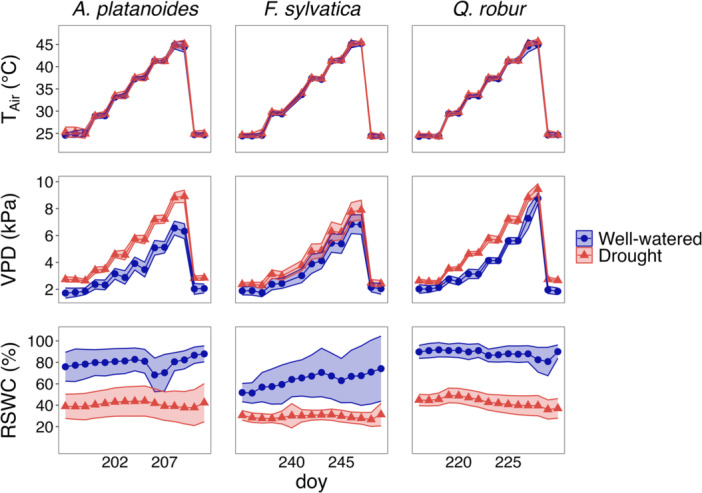
Environmental conditions during the heat experiment per treatment for *A. platanoides, Q. robur* and *F. sylvatica* (*n* = 6–10). Mean daytime (10:00–18:00) values for air temperature (T_air_), vapour pressure deficit (VPD) and relative soil water content (RSWC) are shown per day of the year (doy) with shaded areas representing the standard deviation.

Each species was exposed to a heat experiment lasting for 15 days with an initial 3 days at 25°C and a following stepwise temperature increase every second day to a maximum of 29, 33, 37, 41, 45°C, followed by a 2‐day recovery phase at 25°C (Figure 1, see Supporting Information Table S2 for average temperatures of each temperature step). Night‐time temperatures rose in accordance with daytime temperatures, with a 0.5°C increase in night‐time temperature for every 1°C increase in daytime temperature (see Supporting Information Figure S1), as night temperatures are predicted to increase at lower rates than daytime temperature during heat waves (De Boeck et al. 2010). Pre‐defined air temperatures were targeted for 10 h per day, with a 3 h day/night transition in which temperatures were slowly increased/decreased. VPD increased along with T_air_, simulating realistic heat conditions, as heat waves and high VPD typically cooccur (Grossiord et al. 2020). Note that humidity was provided in a shared incoming air stream, so both treatments received identical supply conditions. Because transpiration rates were typically higher in the well‐watered trees, this resulted in a lower temperature‐driven increase of VPD compared to the drought treatment (see below and Figure 1). Outside light was supplemented by sodium vapour lamps and daytime length set to 16 h. The light conditions among the chambers were monitored and kept constant with PAR of 397 ± 31 µmol m^−2^ s^−1^.

### 2.3 Tree Flux System

For continuous monitoring of above‐ and belowground gas fluxes during the heat experiment, we employed our custom‐made Tree Flux System with 20 individual chambers placed inside a greenhouse compartment (Birami et al. 2020; Rehschuh and Ruehr 2022). Each chamber consists of a transparent, temperature‐controlled shoot compartment and a separate opaque root compartment. The above ‐ and belowground compartments are gas‐tightly separated allowing to measure shoot and root fluxes separately. Air temperature of each aboveground compartment was controlled individually: each chamber was equipped with fast response thermocouples (5SC‐TTTI‐36‐2M, Newport Electronics GmbH, Deckenpfronn, Germany) and a ventilation system (Mini Kaze 60 mm, Scythe, Tokyo, Japan) connected to a cooling system. T_air_ was recorded every 10 s and if it exceeded a pre‐set threshold, the cooling system was activated for each aboveground compartment separately.

Photodiodes (G1118; Hamamatsu Photonics, Hamamatsu, Japan) recorded the PAR at the top of each chamber. In the belowground compartments, soil temperature (T_soil_) was tracked using temperature probes (107, Campbell Scientific Inc. USA) and soil water content (SWC) was monitored with soil moisture sensors (ECH20 10HS, Meter Group, USA). Water addition to each chamber was controlled through an automated drip irrigation system. PAR, T_soil_ and SWC were logged every 10 min (CR1000, Campbell Scientific Inc. USA).

Gas exchange was monitored with a measurement system alternating between chambers. For each of the individual shoot and root compartments, inward (Air_supply_) and outward fluxes (Air_sample_) for water (mmol H_2_O mol air^‐1^) and carbon dioxide (ppm) were recorded every 80, 85 or 100 min for *A. platanoides, Q. robur* and *F. sylvatica*, respectively, thereby enabling the reconstruction of diurnal gas exchange patterns. Note that recording intervals differ, because a different number of chambers was utilised for each species‐specific run. Each recording per chamber compartment took 150 s with data being logged every 10 s, resulting in 15 data points per measurement period per compartment. All chamber‐based measurements and time stamps are reported in Central European Time (CET). Air_supply_ into the chambers was set to a CO_2_ concentration of 432 ± 15 ppm and H_2_O concentration of 1.4 ± 0.2 mmol H_2_O mol air^‐1^. Alongside increasing air temperatures, this constant H_2_O concentration, resulted in an increase of VPD inside the chambers, which was slightly lower in the well‐watered seedlings due to higher transpiration rates. Flow rates for Air_supply_ were adjusted to each tree chamber individually with aboveground compartments receiving on average 7.3 l min^‐1^ and belowground compartments 2.6 l min^‐1^. Absolute concentrations of H_2_O and CO_2_ in Air_supply_ were measured before entering the chambers (Li‐840, LI‐COR, Lincoln, NE, USA). In addition, a differential gas analyser was used to measure the difference between Air_supply_ and Air_sample_ (Li‐7000, LI‐COR Lincoln, NE, USA), using the absolute concentrations in Air_supply_ from the Li‐840 as reference values. For further information on the technical details of the Tree Flux system see Supporting Information Figure S2 and Birami et al. (2020) or Rehschuh et al. (2022).

To eliminate any off‐sets in CO_2_ and H_2_O fluxes not caused by plant gas exchange, an empty chamber was measured in each experimental run. Measuring differences between Air_supply_ and Air_sample_ were detected at on average 1.9 ± 1.4 ppm CO_2_ and 0.06 ± 0.08 ppm H_2_O in the shoot compartments. Respective offsets were removed accordingly before further proceeding with the gas exchange calculations.

The concentration differences between Air_supply_ and Air_sample_ and the chamber‐specific flow rates were used to calculate CO_2_ and H_2_O gas‐exchange fluxes, following the equation summary of the LI‐6400/XT Portable Photosynthesis System. Gas exchange fluxes were scaled to leaf area in m^2^.

Transpiration *E* (mol m^‐2^ s^‐1^) was derived as follows:

$$E\;=\;\frac{F_{\text{in}}\;\cdot \;{(W}_{\mathrm{sample}}-\;W_{\mathrm{supply}})}{\mathrm{LA}\;\cdot \;(1-\;W_{\mathrm{sample}})},$$

where F_in_ (mol s^‐1^) equals the air mass flow into each chamber W_sample_ (mol H_2_O mol air^‐1^) is the H_2_O vapour concentration of Air_sample_, W_supply_ (mol H_2_O mol air^‐1^) is the H_2_O vapour concentration of Air_supply_ and LA is the leaf area per tree (m^2^).

The CO_2_ fluxes for calculating A_net_ (mol m^‐2^ s^‐1^) were determined by:

$${\mathrm{CO}}_{2}\;\mathrm{flux}\;=\;-\frac{F_{\text{in}}\left(C_{\mathrm{sample}}-C_{\mathrm{supply}}\right)}{\mathrm{LA}}\;-(C_{\mathrm{sample}}\;\cdot \;E),$$

where C_sample_ (mol CO_2_ mol air^‐1^) is the CO_2_ concentration of Air_sample_, C_supply_ (mol CO_2_ mol air^‐1^) is the CO_2_ concentration of Air_supply_. Measurements were corrected for dilution by water vapour *E* (mol s^‐1^).

In addition, stomatal conductance g_s_ (mol m^‐2^ s^‐1^) was calculated as follows:

$$g_{s}\;=\;\frac{E\;\cdot (1-\;\frac{W_{\mathrm{leaf}}\;+\;W_{\mathrm{sample}}}{2})}{W_{\mathrm{leaf}\;}-\;W_{\mathrm{sample}}},$$

where W_leaf_ is the H_2_O vapour concentration of the leaf, obtained from the saturation vapour pressure (kPa) at the given leaf temperature (°C) and atmospheric pressure (kPa). This method assumes well‐mixed air conditions and neglects the boundary‐layer conductance, as found in the aboveground compartments with a steady in‐ and outward flow and constant ventilation.

Apparent water‐use efficiency WUE_a_ was derived as:

$${\mathrm{WUE}}_{a}\;=\;\frac{A_{\mathrm{net}}}{E}.$$

Finally, intrinsic water‐use efficiency WUE_i_ was calculated as follows:

$${\mathrm{WUE}}_{i}\;=\;\frac{A_{\mathrm{net}}}{g_{s}}.$$

### 2.4 Biomass Sampling and Leaf Senescence

At the end of the experiment, tree biomass was harvested and split into roots, stem, damaged leaves (> 50% visually affected leaf area) and undamaged leaves. Leaves were classified as damaged only when more than 50% of the leaf area showed visible necrosis or scorching, whereas leaves with minor or marginal damage were included in the undamaged category. Leaf area for each tree was assessed using a leaf‐area‐metre (Li‐3100C, LI‐COR Inc.). Leaf biomass was assessed after drying at 60°C for 48 h. Leaf biomass and leaf area did not increase due to growth during the short interval of the experiment, but decreased due to stress‐induced leaf senescence which needed to be accounted for as gas exchange was scaled to leaf area. To approximate leaf area loss during the experiment and to scale gas exchange to the approximated leaf area, rather than assuming steady leaf area, a linear model was fit between the initial leaf biomass at the first day of observed damage and the undamaged leaf biomass found at the end of the experiment for each tree. In addition, leaf damage (%) was calculated as the ratio between biomass of damaged leaves after the heat experiment and initial biomass (healthy + damaged biomass at the end of the experiment).

### 2.5 Leaf Temperature

Leaf temperature (T_leaf_) was assessed via thermography using an infra‐red camera (PI 450, Optris, Germany). On the final day of each temperature step, measurements were taken between 10:00 and 12:00 (CET) on the upper canopy leaves of each tree (*n* = 6 and 7 for A. *platanoides*, 7 and 7 for *Q. robur*, and 9 and 10 for *F. sylvatica* per drought and well‐watered treatment, respectively). Mean T_leaf_ was determined using the manufacturer's software (Optris PIX connect). Background radiation was corrected for by the respective T_air_ of the measurement day and emissivity was set to 0.97.

### 2.6 Water Potential

Water potential was measured midday between 12:00 and 14:00 (CET) for 6 individuals per species and treatment. For *A. platanoides*, entire leaves were sampled and midday water potential determined using a Scholander pressure chamber (Model 1000, PMS Instruments, OR, USA), as latex in the petioles interfered with thermocouple psychrometer measurements. For *Q. robur* and *F. sylvatica*, leaf discs (6 mm diameter) were excised from individual leaves and measured using C52‐psychrometers connected to a PSYPRO water potential system (Wescor, Logan, UT, USA) to minimise destructive sampling, as pressure chamber measurements for those two species would have required harvesting entire shoots rather than individual leaves.

### 2.7 Data Processing and Analysis

All data processing and statistical analysis was conducted using R version 4.3.1 (R Core Team 2022). Prior to the analysis, all gas exchange data was inspected visually per chamber. Sequences where technical errors occurred were removed (a total of 14, 12 and 27 h for *A. platanoides, Q. robur* and *F. sylvatica*, respectively). Since the gas analyser required time to stabilise after switching between chambers, for each measurement period per chamber only the last five values out of the 15 measurements were used to derive the mean gas exchange for the measurement period.

To test for the effect of the drought treatment and T_air_ and their interaction on T_leaf_, we applied generalised linear mixed models (glmmTMB, version 1.1.9; Brooks et al. 2017) for each species. Individual gas exchange chambers were included as a random intercept, and heteroscedasticity across treatments was accounted for if significant. To evaluate the treatment difference at intermediate heat stress, T_air_ was scaled to 45°C. The same model approach was applied for analysing the relationship between E and T_diff_. For visualisation, we additionally show the treatment‐averaged marginal fitted relationship between E and T_diff_.

The influence of the drought treatment and increasing T_air_ on all gas exchange variables (A_net_, g_s_, *E*, WUE_a_, WUE_i_) was assessed using generalised additive models (mgcv, version 1.8; Wood 2023) to allow for nonlinear relationships between air temperature and gas exchange. Models were fitted per species, with chamber included as a random intercept. Smooth terms for air temperature were fitted separately for each treatment using a basis dimension of k = 10, and effective degrees of freedom were estimated by REML. To meet model assumptions of normality and homoscedasticity of residuals, g_s_ for *A. platanoides* was log‐transformed prior to analysis. Regression coefficients for g_s_ for this species are therefore reported on the log scale. Post‐hoc comparisons were performed using the emmeans package (version 1.8.8; Lenth 2023), applying Tukey‐adjusted pairwise contrasts to estimate differences between treatments at each temperature step and differences among temperature steps within each treatment. Estimated marginal means and contrasts are presented on the response scale.

To assess treatment differences in leaf area loss, which was only evaluated once at the end of the experiment, we applied linear models per species. Finally, recovery of gas exchange was analysed using generalised linear mixed models (glmmTMB, version 1.1.9, Brooks et al. 2017) with chamber included as a random intercept, to test for differences in A_net_ and g_s_ between pre‐stress (25°C), stress (45°C) and post‐heat recovery day 1 (R1, 25°C) and day 2 (R2, 25°C) within each species. Again, estimated marginal means were obtained using the emmeans package and Tukey‐adjusted pairwise comparisons were used.

Throughout the results, values are reported as model‐estimated means ± standard error, based on the respective statistical model fitted for each response variable. Relative changes are reported as percentage differences derived from these model estimates.

## 3 RESULTS

### 3.1 Gas Exchange

**Figure 2:**
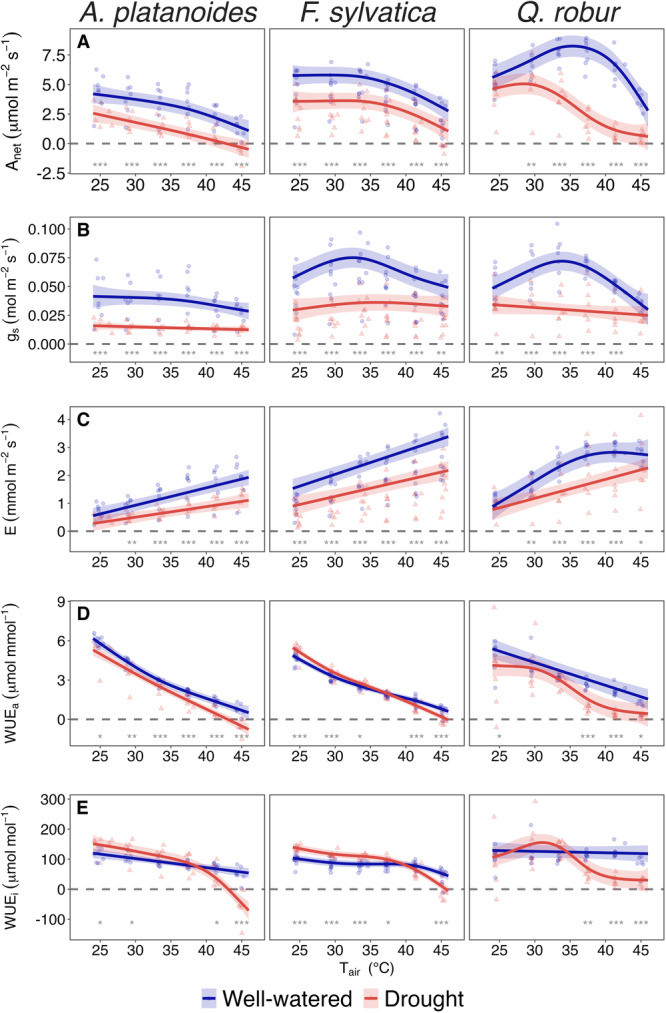
Gas exchange dynamics with increasing air temperature in *A. platanoides, F. sylvatica and Q. robur*. Mean daytime (10:00–18:00) values are shown for net assimilation A_net_, stomatal conductance g_s_, transpiration *E*, apparent water use efficiency WUE_a_ and intrinsic water use efficiency WUE_i_. Points show mean values per tree and temperature step (*n* = 6–10 trees per species and treatment). Lines represent generalised additive mixed models with 95% confidence interval per treatment. Asterisks indicate significant treatment differences at each temperature step (*p < 0.05, **p < 0.01, ***p < 0.001). [Color figure can be viewed at wileyonlinelibrary.com]

In all tree species, leaf gas exchange was directly affected by increasing air temperatures along increasing VPD under both well‐watered and moderate drought conditions (Figure 2, Table 1). However, combined heat and drought exerted the strongest effects, with clear species‐specific differences.

**Table 1:** Summary of statistical effects of air temperature (T_air_), drought treatment, and their interaction (T_air_ × drought), as well as transpiration (*E*) or recovery stage where applicable, on leaf gas exchange, leaf temperature, and damage across the three studied species (*A. platanoides*, *F. sylvatica*, *Q. robur*). Traits are grouped according to the hypotheses they address. Reported are test statistics and associated *p*‐values derived from the respective models: F‐statistics for smooth terms in GAMs and linear models, t‐values for parametric terms in GAMs, and χ² statistics for mixed‐effects models fitted with glmmTMB (Type III Wald tests). Interactions in GAMs were tested using likelihood ratio tests and are reported as *p*‐values only.

Trait	Linked hypothesis	Effect	*A. platanoides*	*F. sylvatica*	*Q. robur*
A_net_ (µmol m⁻² s⁻¹)	1,2	T_air_	F = 11.6, *p* < 0.001	F = 11.6, *p* < 0.001	F = 2.7, *p* = 0.1
		Drought	t = −7.9, *p* < 0.001	t = −12.9, *p* < 0.001	t = −12.9, *p* < 0.001
		T_air_ x Drought	NA	*p* < 0.37	*p* < 0.001
g_s_ (mol m⁻² s⁻¹)	1,2	T_air_	F = 1.36, *p* = 0.248	F = 1.61, *p* = 0.264	F = 0.3, *p* = 0.607
		Drought	t = −11.8, *p* < 0.001	t = −10.9, *p* < 0.001	t = −9.1, *p* < 0.001
		T_air_ x Drought	*p* = 0.411	*p* = 0.060	*p* < 0.001
*E* (mmol m⁻² s⁻¹)	1,2	T_air_	F = 51.5, *p* < 0.001	F = 28.2, *p* < 0.001	F = 27.0, *p* < 0.001
		Drought	t = 6.1, *p* < 0.001	t = −8.86, *p* < 0.001	t = −5.23, *p* < 0.001
		T_air_ x Drought	*p* = 0.06	*p* = 0.08	*p* = 0.024
WUE_a_ (µmol mmol⁻¹)	1,2	T_air_	F = 22.7, *p* < 0.001	F = 181.4, *p* < 0.001	F = 0.1, *p* = 0.756
		Drought	t = 32.6, *p* < 0.001	t = 0.1, *p* = 0.7	t = 22.3, *p* < 0.001
		T_air_ x Drought	*p* = 0.333	*p* < 0.001	*p* = 0.073
WUE_i_ (µmol mmol⁻¹)	1,2	T_air_	F = 9.3, *p* = 0.003	F = 9.1, *p* < 0.001	F = 0.15, *p* = 0.701
		Drought	t = −1.1, *p* = 0.257	t = 2.43, *p* = 0.02	t = 3.78, *p* < 0.001
		T_air_ x Drought	*p* < 0.001	*p* < 0.001	*p* < 0.001
T_leaf_ (°C)	1,2	T_air_	χ² = 274.4, *p* < 0.001	χ² = 941.8, *p* < 0.001	χ² = 912.6, *p* < 0.001
		Drought	χ² = 2.2, *p* = 0.14	χ² = 10.4, *p* = 0.001	χ² = 18.39, *p* < 0.001
		T_air_ x Drought	χ² = 8.9, *p* = 0.002	χ² = 7.78, *p* = 0.005	χ² = 45.5, *p* < 0.001
T_diff_ (°C)	1,2	*E*	χ² = 4.6, *p* = 0.031	χ² = 21.3, *p* < 0.001	χ² = 28, *p* < 0.001
		Drought	χ² = 0.6, *p* = 0.44	χ² = 0.03, *p* = 0.857	χ² = 2.75, *p* = 0.097
		*E* x Drought	χ² = 0.08, *p* = 0.77	χ² = 1.5, *p* = 0.224	χ² = 21.4, *p* < 0.001
Leaf damage (%)	3	Drought	F = 33.9, *p* < 0.001	F = 5.74, *p* = 0.028	—
Recovery A_net_ (% pre‐stress)	3	Recovery step	χ² = 823.8, *p* < 0.001	χ² = 295.6, *p* < 0.001	χ² = 77.9, *p* < 0.001
		Drought	χ² = 37.1, *p* < 0.001	χ² = 17.3, *p* < 0.001	χ² = 3.9, *p* = 0.049
Recovery g_s_ (% pre‐stress)	3	Recovery step	χ² = 10.4, *p* = 0.015	χ² = 17.4, *p* < 0.001	χ² = 17.1, *p* < 0.001
		Drought	χ² = 43.8, *p* < 0.001	χ² = 28.2, *p* < 0.001	χ² = 6.9, *p* = 0.009

In the well‐watered treatment, mean daytime net assimilation rates (A_net_) only declined slightly up to 37°C for *A. platanoides* and *F. sylvatica* (Figure 2A, Supporting Information Table S5). Beyond this temperature, A_net_ declined more strongly, with values at 45°C being reduced by ca. 70% and 50% relative to 25°C for *A. platanoides* and *F. sylvatica*, respectively. *Q. robur* exhibited higher overall A_net_ which even increased under well‐watered conditions up to 37°C by 2.29 ± 0.58 µmol s^‐1^ m^‐2^ before strongly decreasing at higher temperatures. In the drought treatment, A_net_ was already lower at 25°C in *A. platanoides* and *F. sylvatica* (reductions compared to well‐watered of 1.7 ± 0.47 and 2.19 ± 0.48 µmol s^‐1^ m^‐2^, respectively, Supporting Information Table S4) and declined further with increasing temperature (Supporting Information Table S5). In *Q. robur*, drought effects were negligible at 25°C but increasingly pronounced above 30°C, leading to a reduction of up to 5.6 ± 0.53 µmol s^‐1^ m^‐2^ compared to the well‐watered treatment at 41°C. Full model statistics (Supporting Information Table S3) and pairwise comparisons for all gas exchange variables per treatment (Supporting Information Table S4) and per temperature step (Supporting Information Table S5) can be found in the supplementary.

Stomatal conductance (g_s_, Figure 2B) in the well‐watered treatment followed largely the shape of A_net_, but increased slightly up to 33°C, most markedly in *Q. robur*. In the drought treatment, g_s_ remained consistently low and showed little temperature effect, with reduction of ca. 50% in A. *platanoides* and *F. sylvatica* and ca. 35% for *Q. robur* compared to the well‐watered treatment at 25°C.

Transpiration (*E*, Figure 2C) increased strongly in all species and treatments with increasing T_air_ and VPD. Highest mean *E* rates were observed at 45°C in *F. sylvatica* (3.30 ± 0.16 mmol m⁻² s⁻¹), followed by *Q. robur* (2.75 ± 0.24 mmol m⁻² s⁻^1^) and *A. platanoides* (1.86 ± 0.13 mmol m⁻² s⁻¹), with *E* in the drought treatment being significantly lower for all species (Tukey pairwise comparisons, *p* < 0.05, Supporting Information Table S4).

Due to the simultaneous reduction in A_net_ and increase in *E* with increasing T_air_, apparent water‐use efficiency (WUE_a_, Figure 2D) sharply declined in all species and treatments (Supporting Information Table S3). In contrast, A_net_ and g_s_ stayed mostly coupled and thus intrinsic water‐use efficiency (WUE_i_, Figure 2E) remained relatively stable with rising T_air_ under well‐watered conditions for *Q. robur* and *F. sylvatica*, while it decreased slightly in *A. platanoides*. Under drought, however, WUE_i_ showed strong reductions between 33°C and 37°C for all species, as g_s_ was already constrained and changed little with further warming, while A_net_ declined (Supporting Information Table S4).

### 3.2 Leaf Temperature Regulation

**Figure 3:**
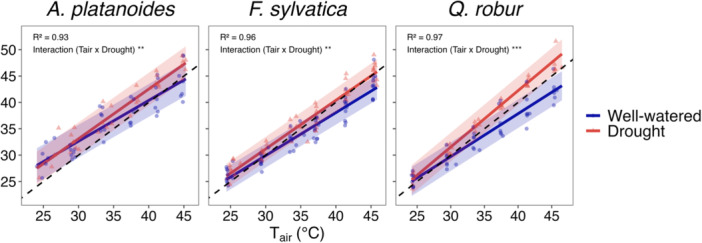
Relationship between leaf (T_leaf_) and air temperature (T_air_) in the well‐watered (blue) and drought treated (red) trees (*n* = 6–10 trees per species and treatment). Points show individual measurements and coloured lines indicate the generalised linear mixed model fitted per species, with shaded areas representing the 95% confidence interval. The black dotted line shows the 1:1 relationship. Asterisks indicate the significance of the interaction term in the model (*p < 0.05, **p < 0.01, ***p < 0.001). [Color figure can be viewed at wileyonlinelibrary.com]

We found leaf temperature (T_leaf_) to increase linearly with T_air_, but with different magnitudes among species and treatments (Figure 3, Supporting Information Table S6). In the well‐watered treatment, T_leaf_ remained largely below T_air,_ with *A. platanoides* being the exception. Leaf cooling under drought was clearly hampered and T_leaf_ values were either equal to or higher than T_air_ (see Supporting Information Table S6), with drought treatment exceeding T_leaf_ of well‐watered treatment at 45°C by 5.7 ± 0.8°C for *Q. robur*, 2.9 ± 0.7°C for *F. sylvatica* and 3.0 ± 1.0°C for *A. platanoides*.

**Figure 4:**
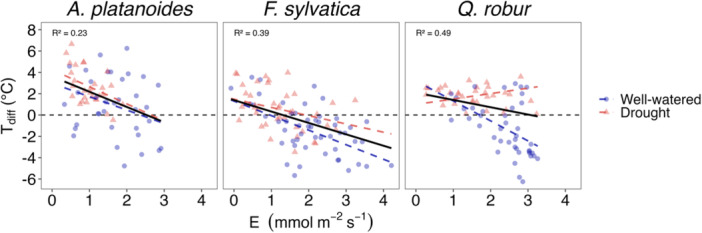
Relationship between transpiration rate (*E*) and leaf‐to‐air temperature difference (T_diff_) for each species. Each data point represents an individual measurement of leaf temperature with the respective transpiration rate given as mean value during 11:00–13:00. Dotted coloured lines show treatment‐specific predictions from the linear mixed‐effects model fit per species, and the black line shows the model prediction averaged across treatments. Reported R^2^ values refer to the full mixed‐effects model including treatment effects. [Color figure can be viewed at wileyonlinelibrary.com]

We found that higher *E* is associated with leaf cooling, resulting in a lower, and even negative leaf‐to‐air temperature difference (T_diff_, Figure 4, Supporting Information Table S7). The strongest cooling effect at a comparable transpiration rate of 2 mmol mol^‐1^ m^‐2^ was found under well‐watered conditions in *F. sylvatica* (T_diff_ = − 1.42 ± 0.4°C) followed by *Q. robur* (T_diff_ = − 0.55 ± 0.57°C), while *A. platanoides* showed on average a net warming (T_diff_ = 0.46 ± 0.61°C). Under drought, the cooling effect of transpiration was diminished, resulting in higher T_diff_, particularly for *Q. robur* (Supporting Information Table S7), indicating a weaker coupling between leaf temperature and transpiration under drought conditions.

### 3.3 Leaf Damage

**Figure 5:**
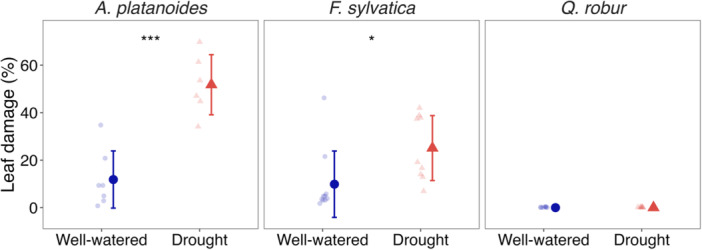
Leaf damage in relation to total leaf area (%) at the end of the experiment for each treatment and species. Shown are mean values (solid colour) and standard deviation, with the lighter points representing individual tree measurements. Asterisks indicate significant differences between treatments (*p < 0.05, **p < 0.01, ***p < 0.001) [Color figure can be viewed at wileyonlinelibrary.com]

The degree of leaf damage measured at the end of the experiment differed markedly between the treatments and tree species (Figure 5). The well‐watered treatment resulted in moderately necrotic leaf tissues, obvious in *A. platanoides* (11.8 ± 4.7%) and *F. sylvatica* (9.9 ± 4.37%, Supporting Information Table S8). Combined with drought much more leaf necrosis was caused, with *A. platanoides* showing the highest leaf damage with 52 ± 12.6%, while *F. sylvatica* depicted only 25 ± 13.6% of leaf damage. In stark contrast, *Q. robur* did not show any leaf shedding or symptoms of wilting (see also Supporting Information Figure S3 for pictures of leaves post‐heat).

### 3.4 Recovery

**Figure 6:**
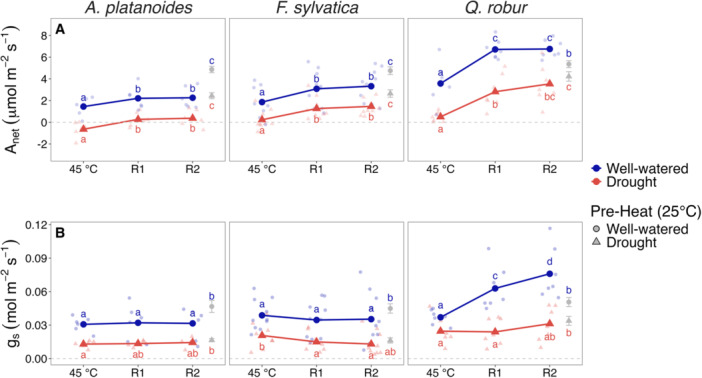
Impacts of heat stress and post‐heat recovery on gas exchange. Shown are mean daytime (10:00–18:00) values of net assimilation rate (A_net_) and stomatal conductance (g_s_) per species and treatment during the final day of heat stress (45°C), and the recovery phase (R1 = one day post heat stress, R2 = two days post heat stress). Solid coloured symbols and lines show treatment mean values, smaller lighter symbols show values per tree, and grey symbols indicate the pre‐heat stress averages (25°C). Different letters indicate significant differences among recovery stages within each treatment. Lowercase blue letters denote comparisons within the well‐watered treatment, whereas uppercase red letters denote comparisons within the drought treatment. [Color figure can be viewed at wileyonlinelibrary.com]

Two days of recovery following heat release only led to minor increases in A_net_ and g_s_ compared to measurements at 45°C for *A. platanoides* and *F. sylvatica* (Figure 6). Importantly, all recovery measurements are expressed per unit remaining leaf area and therefore reflect the physiological performance of intact leaf tissue rather than the loss of leaf area due to heat‐induced senescence. Even on this per‐leaf‐area basis, A_net_ and g_s_ only increased slightly two days after heat release and A_net_ remained below pre‐stress values for A. *platanoides* (by 2.58 ± 0.14 µmol s^‐1^ m^‐2^) and *F. sylvatica* (by 1.39 ± 0.18 µmol s^‐1^ m^‐2^, Supporting Information Table S10). In contrast, *Q. robur* displayed a more pronounced recovery response, with well‐watered seedlings even exceeding pre‐stress values of A_net_ and g_s_ by > 25%.

The post‐heat recovery of the drought treatment indicated a much lower recovery response in all species. In drought‐treated *A. platanoides* and *F. sylvatica*, A_net_ remained well below pre‐stress levels by ca. 70% and 50%, respectively, whereas g_s_ showed almost no difference across pre‐heat, heat stress, and recovery measurements (see Supporting Information Tables S9 and S10). In *Q. robur*, the recovery response was stronger and both A_net_ and g_s_ reached pre‐stress levels, yet not overshooting as in the well‐watered treatment. Over all treatments and species, patterns of recovery paralleled differences in leaf damage, with *Q. robur* showing both, the least leaf damage and highest instantaneous recovery of gas exchange.

## 4 DISCUSSION

Our results demonstrate that increasing heat stress impacted leaf gas exchange, leaf temperature regulation and leaf integrity, with these impacts markedly intensified under moderate water limitation. Species‐specific hydraulic strategies along the isohydric‐anisohydric spectrum influenced the degree to which leaf cooling, assimilation and tissue damage were affected, ultimately shaping the extent of recovery two days post heat stress.

### 4.1 Leaf Temperature Regulation

Transpiration played a central role in leaf temperature regulation under increasing air temperature. T_leaf_ increased with T_air_ across all species. Yet, under well‐watered conditions, T_leaf_ remained significantly below T_air_ (Figure 3), indicating active leaf cooling (Cook et al. 2021; Teskey et al. 2015). As expected, T_diff_ decreased with higher *E* (Figure 4). This direct link demonstrates the thermoregulatory function of *E*, a mechanism often suggested but rarely quantified empirically; but see Drake et al. (2018) and Urban et al. (2017). However, transpirational leaf cooling came at the cost of a pronounced decline in WUE_a_ (Figure 2 D). Interestingly, WUE_i_ responded less to increasing temperature under well‐watered conditions, particularly in *Q. robur*. Thus, A_net_ and g_s_ maintained tightly coupled when water availability was sufficient to meet the increasing evaporative demand. This suggests that rising VPD, rather than stomatal opening (Diao et al. 2024; Marchin et al. 2023), predominantly drove increased *E*; with a potentially stronger effect under drought conditions, where VPD was slightly higher.

### 4.2 Drought Effects on Gas Exchange and Thermal Stress

Limiting water supply modulated the heat stress response, and leaf physiological functioning declined. Because drought intensity was imposed relative to each species' physiological tolerance, our interpretation focuses on within‐species contrasts between well‐watered and drought treatments rather than absolute cross‐species differences in soil moisture. Rising temperatures limited gas exchange and A_net_ dropped to values close to or surpassing zero (Figure 2A), indicating a shift to net respiration (Birami et al. 2020; Marchin et al. 2023), thereby turning trees from carbon sinks into carbon sources (Rehschuh and Ruehr 2022). Despite partial stomatal closure under hot drought, *E* continued to increase with rising T_air_ and VPD in all species (Figure 2C), similar to findings by Bachofen et al. (2025) and Ruehr et al. (2016). Residual and cuticular water loss likely contributed to this continued transpiration flux (Duursma et al. 2019; Slot et al. 2021). As temperatures approached 33°C–37°C, g_s_ and A_net_ decoupled in all species under drought, reflected in a strongly reduced WUE_i_ (Figure 2E). Similar patterns have been reported previously under combined heat and drought stress (Ruehr et al. 2016; Watson‐Lazowski et al. 2025), and are likely the result of heat‐induced biochemical limitations to carbon uptake including Photosytem II impairments (Mathur et al. 2014).

Under drought, T_leaf_ was higher than T_air_ throughout all species (Figure 3), despite continued *E*. This pattern was particularly pronounced in *Q. robur*, where increasing transpiration did not consistently translate into lower leaf temperatures, indicating that transpiration alone cannot fully explain leaf temperature developments under hot drought conditions. These higher leaf temperatures align with findings in e.g. Scots pine (Rehschuh and Ruehr 2022) or Eucalypts (Drake et al. 2018). Higher leaf temperatures can directly translate into greater leaf damage under drought, when critical thresholds are exceeded (Esperon‐Rodriguez et al. 2021; Posch et al. 2024). In our study, hot drought caused significantly higher leaf damage in *A. platanoides* and *F. sylvatica* than heat alone (Figure 5). Typical scorching signs from overheating (Supporting Information Figure S3) along with considerably stable midday water potentials (Supporting Information Figure S4) point towards thermal rather than hydraulic stress as the dominant mechanism. This is consistent with a pronounced decline in F_v_/F_m_ reported for *A. platanoides* and *F. sylvatica* at 45°C (Hauck et al. 2025).

Collectively, these results support our first hypothesis and emphasise that even moderate water limitation during heat stress can substantially impair gas exchange and leaf cooling, increasing vulnerability to thermal damage.

### 4.3 Species Differences and Hydraulic Strategies

Beyond water availability, species‐specific hydraulic strategies and morphological differences contributed to the heat and hot drought response. As anticipated, *A. platanoides* tightly regulated stomata. This resulted in an early decline of A_net_ as well as relatively low transpiration, and thus limited capacity for transpirational cooling (Figures 2A,B and 3). In contrast, midday leaf water potential showed variability among treatments in *A. platanoides* (Supporting Information Figure S4). Nonetheless, the observed early stomatal limitations and reduced transpiration consistently indicate tight stomatal regulation, in agreement with previous classifications of *A. platanoides* as relatively isohydric (Kunz et al. 2016). In addition, the larger leaves of *A. platanoides* likely increased boundary layer resistance, constraining the efficiency of transpirational cooling even at comparable transpiration rates (Pan et al. 2022). Furthermore, *A. platanoides* exhibited the highest leaf damage, consistent with higher specific leaf area and radiative surface (Lin et al. 2017; Monteiro et al. 2016), as well as generally lower leaf thermal tolerance thresholds (Hauck et al. 2025). Moderately anisohydric *F. sylvatica* maintained stomata more open under stress, enabling stronger transpirational cooling. This was reflected in lower T_leaf_ and only moderate declines in gas exchange and leaf area, although sustained transpiration under warming may ultimately reduce drought tolerance by impairing water relations and hydraulic functioning (Rubio et al. 2025). Reported critical temperature values (T5) further support this ranking with *A. platanoides* showing initial thermal damage to Photosystem II at leaf temperatures of 38.1°C, *F. sylvatica* at 40.7°C and *Q. robur* at 44.2°C (Münchinger et al. 2023).

Our study demonstrated that under well‐water conditions, anisohydric *Q. robur* maintained open stomata, sustained or even increased A_net_ up to 37°C, and avoided any major leaf damage. These observations align with previous work showing high thermal thresholds (Gauthey et al. 2024; Hauck et al. 2025). Consistent with this, *Q. robur* also maintained a steeper A_net_ ‐ g_s_ relationship under heat stress, suggesting that at similar g_s_, this species achieves higher A_net_ above 40°C, indicative of efficient photosynthetic function and enhanced thermotolerance (Supporting Information Figure S5).

Importantly, *Q. robur* also showed evidence of dynamic stomatal regulation, with peaks in morning and evening hours when air temperatures and VPD were lower (Supporting Information Figure S6), indicating adaptive regulation under high VPD – a strategy also documented in field studies for *Q. rubra* (Bauweraerts et al. 2014) and *P. sylvestris* (Gauthey et al. 2023). In contrast, other studies have shown that *Q. robur* controls stomata more strictly, reducing transpiration under soil and atmospheric drought (Niemczyk et al. 2024). This might originate from measurements that are restricted to one discrete time point during the day (e.g. midday). In our study however, we have measured gas exchange continuously and thus, provide a more coherent picture.

However, under drought these advantages diminished as declining soil moisture constrained g_s_, A_net_ and transpirational cooling. This is consistent with observations in other anisohydric species, where elevated temperatures and VPD maintained stomatal opening when water was available, but reduced soil moisture clearly negated this response (Grossiord et al. 2017).

Overall, our results support the second hypothesis that anisohydric *Q. robur* and *F. sylvatica* exhibit greater heat tolerance than the isohydric *A. platanoides*. Yet, the pronounced tolerance of *Q. robur* indicates that heat tolerance emerges from the combined action of hydraulic strategy, structural leaf traits, and tissue‐level heat tolerance.

### 4.4 Recovery From Heat Stress

Contrary to our expectations, water availability only played a marginal role for determining recovery trajectories of gas exchange rates two days after heat release, while species identity was a dominant factor.

*Q. robur* recovered A_net_ rates to 80% and g_s_ to 90% of pre‐stress levels at 25°C two days after heat release in the drought treatment, and well‐watered seedlings even exceeded pre‐stress rates. This is in line with the observed higher heat tolerance and lower leaf damage in *Q. robur*, its generally high photosynthetic capacity (Didion‐Gency et al. 2022), and previous findings on the superior recovery of oak (Kannenberg et al. 2019). In contrast, *F. sylvatica* and particularly *A. platanoides* showed only limited recovery. In both species, recovery of g_s_ was minimal, even under well‐watered conditions (Figure 6B). The overall minimal variation in g_s_ under drought shows that full stomatal closure had potentially been achieved, leaving little additional regulatory capacity during the heat stress or subsequent recovery.

Water availability did not fundamentally alter recovery trajectories, thereby partly rejecting our third hypothesis. We observed similar dynamics between treatments, just from different baselines. This suggests that the main constraints on recovery were imposed by structural and biochemical heat damages rather than drought‐induced hydraulic impairments. The slightly faster recovery of A_net_ than g_s_ under drought further indicates that non‐stomatal limitations, for example, due to Photosystem II inhibition or impaired metabolic turnover, played a major role for inhibiting and recovering A_net_ (Ruehr et al. 2019). The slower recovery of g_s_ may additionally reflect continued hormonal regulation of stomatal aperture, for example via abscisic acid signalling following combined heat and drought stress (Mittler and Blumwald 2015).

While a two‐day recovery only captures the initial phase of recovery processes (Gessler et al. 2020; Hesse et al. 2023), our results highlight that species‐specific differences in heat damage and necrotic leaf tissue as well as their strategies to restore metabolic functioning – rather than water availability alone – are decisive for recovery. Extending recovery periods and incorporating pro‐longed drought exposure would help disentangle how heat‐induced tissue damage, impaired photosynthetic functioning, stomatal regulation and acclimation interact to constrain recovery under recurring stress.

## 5 Conclusion

Overall, our findings highlight that tree physiological functioning under heat stress strongly depends on water availability and species‐specific hydraulic strategies. Even moderate soil drought strongly weakened the ability of the studied temperate broadleaved tree seedlings to tolerate heat stress by limiting transpirational leaf cooling, causing leaf temperatures to rise above air temperatures, and damaging leaves more severely compared to well‐watered trees.

Anisohydric *Q. robur* sustained high gas exchange rates and fully recovered post‐stress, without any major leaf damage. In contrast, the more isohydric *A. platanoides* and moderately anisohydric *F. sylvatica* exhibited stronger stomatal regulation, higher leaf temperatures and incomplete recovery. These differences illustrate that species‐specific combinations of stomatal regulation, physiological and structural traits shape thermal resistance and short‐term recovery.

Collectively, our results highlight that heat tolerance and recovery in three temperate European tree seedlings depend on the interaction between water availability, transpirational leaf cooling, and species‐specific thermal sensitivity. Incorporating these findings, particularly the rapid loss of leaf cooling under moderate drought, into processed‐based models may improve predictions of leaf thermal stress and post‐heat recovery in forests.

## Conflicts of Interest

The authors declare no conflicts of interest.

## Supporting information


**Figure S1:** Mean night–time (23:00–04:00) air temperatures per treatment for *A. platanoides*, *Q. robur* and *F. sylvatica*. Shaded areas represent ± standard deviation. Gaps in the data originate from system malfunctioning during the night‐time.
**Figure S2:** Schematic overview of the Tree Flux System. Each chamber in the system is separated into above and belowground compartment. Arrows indicate direction of flow. Air supply to the chambers is given in light blue (Air_supply_) and the air returning from the sample is given in dark blue (Air_sample_). Absolute CO_2_ concentrations were measured using the Li‐840 connected to a Li‐7000 measuring differences between Air_supply_ and Air_sample_. Adopted from Birami et al. ( 2018).
**Figure S3:** Visual impressions of leaf damage after heat stress for *A. platanoides* (A, D), *F. sylvatica* (B, E) and *Q. robur* (C, F).
**Figure S4:** Midday Water potential measurements for each species. Big dots show mean values, errorbars show ± standard deviation and light small points representing individual measurement points. Pre and Post refer to measurements taken before and after trees were in the chamber. Note that water potential for *A. platanoides* was measured using a Scholander pressure bomb and for *F. sylvatica* and *Q. robur* using a Psypro.
**Figure S5:** Relationship between net assimilation (A_net_) and stomatal conductance (g_s_) for air temperature < 30°C and > 40°C. Dots show daily mean values per chamber. Lines show linear models fitted for each species and treatment separately.
**Figure S6:** Diurnal dynamics of treatment‐averaged hourly net assimilation (A_net_), canopy conductance (g_s_) and transpiration (E) for each temperature step and species. Lines show treatment means ± standard error (n = 6‐10 per treatment and species). Lighter dots depict individual measurements per tree.
**Table S1:** Total leaf biomass and leaf area at the start of the experiment and damaged leaf biomass at the end of the experiment per treatment and species (mean ± standard deviation).
**Table S2:** Mean temperatures ± standard deviation for each temperature step and species during the heat experiment from 10:00 to 18:00.
**Table S3:** Parametric coefficients from species‐specific generalized additive models (mgcv) describing gas‐exchange responses to air temperature and watering treatment. Note that coefficients for g_s_ in *A. platanoides* are reported on the log scale to meet model assumptions.
**Table S4:** Estimated marginal means and post‐hoc Tukey contrasts for watering treatment effects at each temperature step per species and gas‐exchange variable. Results are obtained from generalized additive models (mgcv) fitted independently for each species and gas‐exchange variable.
**Table S5:** Estimated marginal means and post‐hoc Tukey contrasts between temperature steps per watering treatment, gas‐exchange variable and species. Results are obtained from generalized additive models (mgcv) fitted independently for each species and gas‐exchange variable.
**Table S6:** Parametric coefficients from species‐specific generalized linear mixed models (glmmTMB), assessing the effect of T_air_, drought treatment and their interaction on T_leaf_.
**Table S7:** Parametric coefficients from species‐specific generalized linear mixed models (glmmTMB), assessing the effect of E, drought treatment and their interaction on T_diff_.
**Table S8:** Parametric coefficients from species‐specific linear models, assessing the effect of drought treatment on the amount of leaf damage (%) at the end of the experiment. No model was fitted to *Q. robur*, as no leaf damage was observed.
**Table S9:** Parametric coefficients from species‐specific generalized linear mixed models (glmmTMB) evaluating the effect of different time steps (pre = at 25°C, 45°C = final day of heat stress, R1 = one day post heat stress, R2 = two days post heat stress) and drought treatment on gas‐exchange responses.
**Table S10:** Estimated marginal means and post‐hoc Tukey contrasts at different time steps (pre = at 25°C, 45°C = final day of heat stress, R1 = one day post heat stress, R2 = two days post heat stress) per watering treatment, gas‐exchange variable and species. Results are obtained from generalized linear mixed models (glmmTMB) fitted independently for each species and gas‐exchange variable.

## Data Availability

The data that support the findings of this study are available upon request.
