# Supplementary material for: Water Availability and Hydraulic Strategies Control Leaf Thermoregulation and Damage During Heat Stress and Recovery in Temperate Tree Species
Source: Plant Cell Environ. 2026 May 17;49(9):6296–310. doi: 10.1111/pce.70605 (PMC13436522; doi:10.1111/pce.70605)
Supplement: Supplementary file 1 [file PCE-49-6296-s001.docx]

Supplement

**Figure S1** Mean night-time (23:00-04:00) air temperatures per treatment for *A. platanoides, Q. robur* and *F. sylvatica*. Shaded areas represent ± standard deviation. Gaps in the data originate from system malfunctioning during the night-time.

**Figure S2** Schematic overview of the Tree Flux System. Each chamber in the system is separated into above and belowground compartment. Arrows indicate direction of flow. Air supply to the chambers is given in light blue (Air_supply_) and the air returning from the sample is given in dark blue (Air_sample_). Absolute CO_2_ concentrations were measured using the Li-840 connected to a Li-7000 measuring differences between Air_supply_ and Air_sample_. Adopted from Birami et al. (2018).

A

B

C

DD

F

EE

**Figure S3** Visual impressions of leaf damage after heat stress for *A. platanoides* (A, D), *F. sylvatica* (B, E) and *Q. robur* (C, F).

**Figure S4** Midday Water potential measurements for each species. Big dots show mean values, errorbars show ± standard deviation and light small points representing individual measurement points. Pre and Post refer to measurements taken before and after trees were in the chamber. Note that water potential for *A. platanoides* was measured using a Scholander pressure bomb and for *F. sylvatica* and *Q. robur* using a Psypro.

**Figure S5** Relationship between net assimilation (A_net_) and stomatal conductance (g_s_) for air temperature < 30°C and > 40 °C. Dots show daily mean values per chamber. Lines show linear models fitted for each species and treatment separately.

**Figure S6** Diurnal dynamics of treatment-averaged hourly net assimilation (A_net_), canopy conductance (g_s_) and transpiration (E) for each temperature step and species. Lines show treatment means ± standard error (n = 6-10 per treatment and species). Lighter dots depict individual measurements per tree.

**Table S1** Total leaf biomass and leaf area at the start of the experiment and damaged leaf biomass at the end of the experiment per treatment and species (mean ± standard deviation).

|  | ***A. platanoides*** | | ***F. sylvatica*** | | ***Q. robur*** | |
| --- | --- | --- | --- | --- | --- | --- |
|  | **Well-watered** | **Drought** | **Well-watered** | **Drought** | **Well-watered** | **Drought** |
| Leaf biomass  (g DW) | 11.57 ±  6.85 | 5.17 ±  3.21 | 6.32 ± 1.24 | 3.63 ± 0.84 | 6.32 ± 2.47 | 2.33 ± 0.81 |
| Leaf area  (m^2^) | 0.102 ± 0.04 | 0.042 ± 0.026 | 0.052 ± 0.016 | 0.039 ± 0.016 | 0.052 ± 0.014 | 0.018 ± 0.014 |
| Damaged leaf biomass  (g DW) | 0.98 ± 1.22 | 1.63 ± 1.02 | 0.25 ± 0.29 | 0.5 ± 0.31 | 0 ± 0 | 0 ± 0 |

**Table S2** Mean temperatures ± standard deviation for each temperature step and species during the heat experiment from 10:00 to 18:00.

|  |  |  |  |  |  |  |  |
| --- | --- | --- | --- | --- | --- | --- | --- |
| Species | 25°C | 29°C | 33°C | 37°C | 41°C | 45°C |  |
| *A. platanoides* | 24.89 ± 0.8 | 29.02 ± 0.58 | 33.43 ± 0.61 | 37.48 ± 0.58 | 41.3 ± 0.48 | 44.79 ± 0.94 |  |
| *F. sylvatica* | 24.48 ± 0.55 | 29.55 ± 0.46 | 33.85 ± 0.58 | 37.29 ± 0.46 | 41.37 ± 0.5 | 45.19 ± 0.56 |  |
| *Q. robur* | 24.54 ± 0.52 | 29.59 ± 0.48 | 33.51 ± 0.41 | 37.37 ± 0.49 | 41.31 ± 0.39 | 45.07 ± 0.43 |  |

**Table S3** Parametric coefficients from species-specific generalized additive models (mgcv) describing gas-exchange responses to air temperature and watering treatment. Note that coefficients for g_s_ in *A. platanoides* are reported on the log scale to meet model assumptions.

A. platanoides

|  | **A_net_** | **g_s_** | **E** | **WUEₐ** | **WUEᵢ** |
| --- | --- | --- | --- | --- | --- |
| Intercept | 2.973 *** | -3.304 *** | 1.251 *** | 2.875 *** | 86.538 *** |
|  | (0.246) | (0.056) | (0.101) | (0.091) | (4.501) |
| Drought | -1.905 *** | -0.961 *** | -0.554 *** | -0.760 *** | -7.567 |
|  | (0.243) | (0.082) | (0.091) | (0.134) | (6.629) |
| N | 78 | 78 | 78 | 78 | 78 |
| logLik | -111.194 | 260.462 | -34.427 | -66.249 | -370.428 |
| AIC | 237.388 | -508.974 | 80.624 | 148.824 | 756.791 |

F. sylvatica

|  | **A_net_** | **g_s_** | **E** | **WUEₐ** | **WUEᵢ** |
| --- | --- | --- | --- | --- | --- |
| Intercept | 4.923 *** | 0.064 *** | 2.483 *** | 2.442 *** | 81.123 *** |
|  | (0.281) | (0.003) | (0.130) | (0.078) | (3.861) |
| Drought | -1.978 *** | -0.030 *** | -0.929 *** | 0.025 | 8.189 * |
|  | (0.227) | (0.003) | (0.105) | (0.068) | (3.401) |
| N | 114 | 114 | 114 | 114 | 114 |
| logLik | -178.859 | 324.758 | -92.241 | -40.419 | -486.298 |
| AIC | 377.608 | -630.124 | 196.503 | 104.118 | 997.341 |

Q. robur

|  | **A_net_** | **g_s_** | **E** | **WUEₐ** | **WUEᵢ** |
| --- | --- | --- | --- | --- | --- |
| Intercept | 6.585 *** | 0.057 *** | 2.212 *** | 3.433 *** | 123.643 *** |
|  | (0.317) | (0.003) | (0.142) | (0.283) | (10.296) |
| Drought | -3.594 *** | -0.027 *** | -0.683 *** | -1.132 *** | -33.904 *** |
|  | (0.278) | (0.003) | (0.130) | (0.240) | (8.964) |
| N | 84 | 84 | 84 | 84 | 84 |
| logLik | -133.131 | 245.353 | -71.701 | -122.073 | -425.985 |
| AIC | 290.262 | -474.238 | 159.196 | 261.530 | 870.658 |
| *** p < 0.001; ** p < 0.01; * p < 0.05. | | | | | |

**Table S4** Estimated marginal means and post-hoc Tukey contrasts for watering treatment effects at each temperature step per species and gas-exchange variable. Results are obtained from generalized additive models (mgcv) fitted independently for each species and gas-exchange variable.

| Variable | Species | Temperature (°C) | Comparison | Estimate | Std. Error | p-value |
| --- | --- | --- | --- | --- | --- | --- |
| *A*_net_ | *A. platanoides* | 25 | (Well-watered) - Drought | 1.696 | 0.471 | < 0.001 |
| *A*_net_ | *A. platanoides* | 29 | (Well-watered) - Drought | 1.903 | 0.347 | < 0.001 |
| *A*_net_ | *A. platanoides* | 33 | (Well-watered) - Drought | 2.071 | 0.321 | < 0.001 |
| *A*_net_ | *A. platanoides* | 37 | (Well-watered) - Drought | 2.128 | 0.315 | < 0.001 |
| *A*_net_ | *A. platanoides* | 41 | (Well-watered) - Drought | 1.967 | 0.333 | < 0.001 |
| *A*_net_ | *A. platanoides* | 45 | (Well-watered) - Drought | 1.660 | 0.477 | < 0.001 |
| *A*_net_ | *F. sylvatica* | 25 | (Well-watered) - Drought | 2.192 | 0.483 | < 0.001 |
| *A*_net_ | *F. sylvatica* | 29 | (Well-watered) - Drought | 2.188 | 0.390 | < 0.001 |
| *A*_net_ | *F. sylvatica* | 33 | (Well-watered) - Drought | 2.088 | 0.372 | < 0.001 |
| *A*_net_ | *F. sylvatica* | 37 | (Well-watered) - Drought | 1.923 | 0.361 | < 0.001 |
| *A*_net_ | *F. sylvatica* | 41 | (Well-watered) - Drought | 1.820 | 0.366 | < 0.001 |
| *A*_net_ | *F. sylvatica* | 45 | (Well-watered) - Drought | 1.727 | 0.480 | < 0.001 |
| *A*_net_ | *Q. robur* | 25 | (Well-watered) - Drought | 1.091 | 0.600 | 0.073 |
| *A*_net_ | *Q. robur* | 29 | (Well-watered) - Drought | 1.881 | 0.576 | 0.002 |
| *A*_net_ | *Q. robur* | 33 | (Well-watered) - Drought | 3.808 | 0.525 | < 0.001 |
| *A*_net_ | *Q. robur* | 37 | (Well-watered) - Drought | 5.606 | 0.522 | < 0.001 |
| *A*_net_ | *Q. robur* | 41 | (Well-watered) - Drought | 5.649 | 0.525 | < 0.001 |
| *A*_net_ | *Q. robur* | 45 | (Well-watered) - Drought | 3.038 | 0.653 | < 0.001 |
| *g*_s_ | *A. platanoides* | 25 | (Well-watered) - Drought | 0.026 | 0.005 | < 0.001 |
| *g*_s_ | *A. platanoides* | 29 | (Well-watered) - Drought | 0.026 | 0.003 | < 0.001 |
| *g*_s_ | *A. platanoides* | 33 | (Well-watered) - Drought | 0.025 | 0.003 | < 0.001 |
| *g*_s_ | *A. platanoides* | 37 | (Well-watered) - Drought | 0.024 | 0.003 | < 0.001 |
| *g*_s_ | *A. platanoides* | 41 | (Well-watered) - Drought | 0.021 | 0.003 | < 0.001 |
| *g*_s_ | *A. platanoides* | 45 | (Well-watered) - Drought | 0.017 | 0.004 | < 0.001 |
| *g*_s_ | *F. sylvatica* | 25 | (Well-watered) - Drought | 0.030 | 0.006 | < 0.001 |
| *g*_s_ | *F. sylvatica* | 29 | (Well-watered) - Drought | 0.038 | 0.005 | < 0.001 |
| *g*_s_ | *F. sylvatica* | 33 | (Well-watered) - Drought | 0.039 | 0.004 | < 0.001 |
| *g*_s_ | *F. sylvatica* | 37 | (Well-watered) - Drought | 0.032 | 0.004 | < 0.001 |
| *g*_s_ | *F. sylvatica* | 41 | (Well-watered) - Drought | 0.024 | 0.004 | < 0.001 |
| *g*_s_ | *F. sylvatica* | 45 | (Well-watered) - Drought | 0.018 | 0.006 | 0.002 |
| *g*_s_ | *Q. robur* | 25 | (Well-watered) - Drought | 0.018 | 0.006 | 0.003 |
| *g*_s_ | *Q. robur* | 29 | (Well-watered) - Drought | 0.031 | 0.005 | < 0.001 |
| *g*_s_ | *Q. robur* | 33 | (Well-watered) - Drought | 0.041 | 0.004 | < 0.001 |
| *g*_s_ | *Q. robur* | 37 | (Well-watered) - Drought | 0.039 | 0.004 | < 0.001 |
| *g*_s_ | *Q. robur* | 41 | (Well-watered) - Drought | 0.026 | 0.005 | < 0.001 |
| *g*_s_ | *Q. robur* | 45 | (Well-watered) - Drought | 0.009 | 0.006 | 0.142 |
| *E* | *A. platanoides* | 25 | (Well-watered) - Drought | 0.304 | 0.160 | 0.062 |
| *E* | *A. platanoides* | 29 | (Well-watered) - Drought | 0.402 | 0.121 | 0.001 |
| *E* | *A. platanoides* | 33 | (Well-watered) - Drought | 0.501 | 0.095 | < 0.001 |
| *E* | *A. platanoides* | 37 | (Well-watered) - Drought | 0.599 | 0.094 | < 0.001 |
| *E* | *A. platanoides* | 41 | (Well-watered) - Drought | 0.698 | 0.118 | < 0.001 |
| *E* | *A. platanoides* | 45 | (Well-watered) - Drought | 0.797 | 0.156 | < 0.001 |
| *E* | *F. sylvatica* | 25 | (Well-watered) - Drought | 0.657 | 0.187 | < 0.001 |
| *E* | *F. sylvatica* | 29 | (Well-watered) - Drought | 0.763 | 0.142 | < 0.001 |
| *E* | *F. sylvatica* | 33 | (Well-watered) - Drought | 0.869 | 0.111 | < 0.001 |
| *E* | *F. sylvatica* | 37 | (Well-watered) - Drought | 0.974 | 0.108 | < 0.001 |
| *E* | *F. sylvatica* | 41 | (Well-watered) - Drought | 1.079 | 0.135 | < 0.001 |
| *E* | *F. sylvatica* | 45 | (Well-watered) - Drought | 1.184 | 0.179 | < 0.001 |
| *E* | *Q. robur* | 25 | (Well-watered) - Drought | 0.194 | 0.251 | 0.442 |
| *E* | *Q. robur* | 29 | (Well-watered) - Drought | 0.531 | 0.200 | 0.010 |
| *E* | *Q. robur* | 33 | (Well-watered) - Drought | 0.863 | 0.179 | < 0.001 |
| *E* | *Q. robur* | 37 | (Well-watered) - Drought | 1.024 | 0.177 | < 0.001 |
| *E* | *Q. robur* | 41 | (Well-watered) - Drought | 0.898 | 0.189 | < 0.001 |
| *E* | *Q. robur* | 45 | (Well-watered) - Drought | 0.559 | 0.263 | 0.037 |
| *WUE*_a_ | *A. platanoides* | 25 | (Well-watered) - Drought | 0.796 | 0.282 | 0.006 |
| *WUE*_a_ | *A. platanoides* | 29 | (Well-watered) - Drought | 0.550 | 0.211 | 0.011 |
| *WUE*_a_ | *A. platanoides* | 33 | (Well-watered) - Drought | 0.463 | 0.206 | 0.028 |
| *WUE*_a_ | *A. platanoides* | 37 | (Well-watered) - Drought | 0.615 | 0.202 | 0.003 |
| *WUE*_a_ | *A. platanoides* | 41 | (Well-watered) - Drought | 0.895 | 0.201 | < 0.001 |
| *WUE*_a_ | *A. platanoides* | 45 | (Well-watered) - Drought | 1.201 | 0.291 | < 0.001 |
| *WUE*_a_ | *F. sylvatica* | 25 | (Well-watered) - Drought | -0.549 | 0.149 | < 0.001 |
| *WUE*_a_ | *F. sylvatica* | 29 | (Well-watered) - Drought | -0.359 | 0.136 | 0.010 |
| *WUE*_a_ | *F. sylvatica* | 33 | (Well-watered) - Drought | -0.224 | 0.127 | 0.082 |
| *WUE*_a_ | *F. sylvatica* | 37 | (Well-watered) - Drought | -0.028 | 0.122 | 0.821 |
| *WUE*_a_ | *F. sylvatica* | 41 | (Well-watered) - Drought | 0.323 | 0.127 | 0.013 |
| *WUE*_a_ | *F. sylvatica* | 45 | (Well-watered) - Drought | 0.595 | 0.149 | < 0.001 |
| *WUE*_a_ | *Q. robur* | 25 | (Well-watered) - Drought | 1.115 | 0.469 | 0.020 |
| *WUE*_a_ | *Q. robur* | 29 | (Well-watered) - Drought | 0.551 | 0.403 | 0.175 |
| *WUE*_a_ | *Q. robur* | 33 | (Well-watered) - Drought | 0.645 | 0.355 | 0.073 |
| *WUE*_a_ | *Q. robur* | 37 | (Well-watered) - Drought | 1.418 | 0.349 | < 0.001 |
| *WUE*_a_ | *Q. robur* | 41 | (Well-watered) - Drought | 1.641 | 0.379 | < 0.001 |
| *WUE*_a_ | *Q. robur* | 45 | (Well-watered) - Drought | 1.247 | 0.475 | 0.010 |
| *WUE*_i_ | *A. platanoides* | 25 | (Well-watered) - Drought | -30.537 | 14.219 | 0.035 |
| *WUE*_i_ | *A. platanoides* | 29 | (Well-watered) - Drought | -26.176 | 11.167 | 0.022 |
| *WUE*_i_ | *A. platanoides* | 33 | (Well-watered) - Drought | -19.559 | 10.638 | 0.070 |
| *WUE*_i_ | *A. platanoides* | 37 | (Well-watered) - Drought | -9.639 | 10.331 | 0.354 |
| *WUE*_i_ | *A. platanoides* | 41 | (Well-watered) - Drought | 25.224 | 10.961 | 0.024 |
| *WUE*_i_ | *A. platanoides* | 45 | (Well-watered) - Drought | 103.328 | 13.878 | < 0.001 |
| *WUE*_i_ | *F. sylvatica* | 25 | (Well-watered) - Drought | -34.649 | 7.600 | < 0.001 |
| *WUE*_i_ | *F. sylvatica* | 29 | (Well-watered) - Drought | -29.749 | 7.337 | < 0.001 |
| *WUE*_i_ | *F. sylvatica* | 33 | (Well-watered) - Drought | -26.901 | 6.858 | < 0.001 |
| *WUE*_i_ | *F. sylvatica* | 37 | (Well-watered) - Drought | -16.751 | 6.437 | 0.011 |
| *WUE*_i_ | *F. sylvatica* | 41 | (Well-watered) - Drought | 10.077 | 6.806 | 0.142 |
| *WUE*_i_ | *F. sylvatica* | 45 | (Well-watered) - Drought | 41.921 | 7.596 | < 0.001 |
| *WUE*_i_ | *Q. robur* | 25 | (Well-watered) - Drought | 15.881 | 17.907 | 0.378 |
| *WUE*_i_ | *Q. robur* | 29 | (Well-watered) - Drought | -19.582 | 16.547 | 0.240 |
| *WUE*_i_ | *Q. robur* | 33 | (Well-watered) - Drought | -21.022 | 14.551 | 0.153 |
| *WUE*_i_ | *Q. robur* | 37 | (Well-watered) - Drought | 44.675 | 14.191 | 0.002 |
| *WUE*_i_ | *Q. robur* | 41 | (Well-watered) - Drought | 82.604 | 15.338 | < 0.001 |
| *WUE*_i_ | *Q. robur* | 45 | (Well-watered) - Drought | 88.192 | 18.055 | < 0.001 |

**Table S5** Estimated marginal means and post-hoc Tukey contrasts between temperature steps per watering treatment, gas-exchange variable and species. Results are obtained from generalized additive models (mgcv) fitted independently for each species and gas-exchange variable.

| Variable | Species | Treatment | Temperature comparison | Estimate | Std. Error | p-value | |
| --- | --- | --- | --- | --- | --- | --- | --- |
| *A*_net_ | *A. platanoides* | Well-watered | 25°C - 45°C | 2.805 | 0.49 | < 0.001 | |
| *A*_net_ | *A. platanoides* | Well-watered | 25°C - 29°C | 0.311 | 0.27 | 0.858 | |
| *A*_net_ | *A. platanoides* | Well-watered | 29°C - 33°C | 0.353 | 0.19 | 0.432 | |
| *A*_net_ | *A. platanoides* | Well-watered | 33°C - 37°C | 0.486 | 0.18 | 0.079 | |
| *A*_net_ | *A. platanoides* | Well-watered | 37°C - 41°C | 0.740 | 0.19 | 0.003 | |
| *A*_net_ | *A. platanoides* | Well-watered | 41°C - 45°C | 0.915 | 0.29 | 0.027 | |
| *A*_net_ | *A. platanoides* | Drought | 25°C - 45°C | 2.770 | 0.51 | < 0.001 | |
| *A*_net_ | *A. platanoides* | Drought | 25°C - 29°C | 0.518 | 0.16 | 0.022 | |
| *A*_net_ | *A. platanoides* | Drought | 29°C - 33°C | 0.522 | 0.13 | 0.002 | |
| *A*_net_ | *A. platanoides* | Drought | 33°C - 37°C | 0.542 | 0.12 | < 0.001 | |
| *A*_net_ | *A. platanoides* | Drought | 37°C - 41°C | 0.579 | 0.13 | < 0.001 | |
| *A*_net_ | *A. platanoides* | Drought | 41°C - 45°C | 0.609 | 0.16 | 0.004 | |
| *A*_net_ | *F. sylvatica* | Well-watered | 25°C - 45°C | 2.686 | 0.47 | < 0.001 | |
| *A*_net_ | *F. sylvatica* | Well-watered | 25°C - 29°C | -0.031 | 0.30 | 1.000 | |
| *A*_net_ | *F. sylvatica* | Well-watered | 29°C - 33°C | 0.099 | 0.23 | 0.998 | |
| *A*_net_ | *F. sylvatica* | Well-watered | 33°C - 37°C | 0.507 | 0.23 | 0.250 | |
| *A*_net_ | *F. sylvatica* | Well-watered | 37°C - 41°C | 0.895 | 0.23 | 0.002 | |
| *A*_net_ | *F. sylvatica* | Well-watered | 41°C - 45°C | 1.215 | 0.32 | 0.004 | |
| *A*_net_ | *F. sylvatica* | Drought | 25°C - 45°C | 2.222 | 0.49 | < 0.001 | |
| *A*_net_ | *F. sylvatica* | Drought | 25°C - 29°C | -0.034 | 0.33 | 1.000 | |
| *A*_net_ | *F. sylvatica* | Drought | 29°C - 33°C | -0.001 | 0.24 | 1.000 | |
| *A*_net_ | *F. sylvatica* | Drought | 33°C - 37°C | 0.342 | 0.24 | 0.709 | |
| *A*_net_ | *F. sylvatica* | Drought | 37°C - 41°C | 0.793 | 0.23 | 0.012 | |
| *A*_net_ | *F. sylvatica* | Drought | 41°C - 45°C | 1.122 | 0.33 | 0.011 | |
| *A*_net_ | *Q. robur* | Well-watered | 25°C - 45°C | 2.128 | 0.65 | 0.018 | |
| *A*_net_ | *Q. robur* | Well-watered | 25°C - 29°C | -1.073 | 0.47 | 0.212 | |
| *A*_net_ | *Q. robur* | Well-watered | 29°C - 33°C | -1.113 | 0.47 | 0.187 | |
| *A*_net_ | *Q. robur* | Well-watered | 33°C - 37°C | -0.104 | 0.44 | 1.000 | |
| *A*_net_ | *Q. robur* | Well-watered | 37°C - 41°C | 1.275 | 0.44 | 0.050 | |
| *A*_net_ | *Q. robur* | Well-watered | 41°C - 45°C | 3.144 | 0.58 | < 0.001 | |
| *A*_net_ | *Q. robur* | Drought | 25°C - 45°C | 4.075 | 0.60 | < 0.001 | |
| *A*_net_ | *Q. robur* | Drought | 25°C - 29°C | -0.283 | 0.44 | 0.988 | |
| *A*_net_ | *Q. robur* | Drought | 29°C - 33°C | 0.814 | 0.41 | 0.350 | |
| *A*_net_ | *Q. robur* | Drought | 33°C - 37°C | 1.695 | 0.39 | < 0.001 | |
| *A*_net_ | *Q. robur* | Drought | 37°C - 41°C | 1.318 | 0.38 | 0.012 | |
| *A*_net_ | *Q. robur* | Drought | 41°C - 45°C | 0.533 | 0.49 | 0.886 | |
| *g*_s_ | *A. platanoides* | Well-watered | 25°C - 45°C | 0.012 | 0.01 | 0.356 |  |
| *g*_s_ | *A. platanoides* | Well-watered | 25°C - 29°C | 0.001 | 0.00 | 1.000 |  |
| *g*_s_ | *A. platanoides* | Well-watered | 29°C - 33°C | 0.001 | 0.00 | 0.998 |  |
| *g*_s_ | *A. platanoides* | Well-watered | 33°C - 37°C | 0.002 | 0.00 | 0.874 |  |
| *g*_s_ | *A. platanoides* | Well-watered | 37°C - 41°C | 0.004 | 0.00 | 0.404 |  |
| *g*_s_ | *A. platanoides* | Well-watered | 41°C - 45°C | 0.004 | 0.00 | 0.460 |  |
| *g*_s_ | *A. platanoides* | Drought | 25°C - 45°C | 0.003 | 0.00 | 0.812 |  |
| *g*_s_ | *A. platanoides* | Drought | 25°C - 29°C | 0.001 | 0.00 | 0.859 |  |
| *g*_s_ | *A. platanoides* | Drought | 29°C - 33°C | 0.001 | 0.00 | 0.836 |  |
| *g*_s_ | *A. platanoides* | Drought | 33°C - 37°C | 0.001 | 0.00 | 0.810 |  |
| *g*_s_ | *A. platanoides* | Drought | 37°C - 41°C | 0.001 | 0.00 | 0.779 |  |
| *g*_s_ | *A. platanoides* | Drought | 41°C - 45°C | 0.001 | 0.00 | 0.744 |  |
| *g*_s_ | *F. sylvatica* | Well-watered | 25°C - 45°C | 0.010 | 0.01 | 0.562 |  |
| *g*_s_ | *F. sylvatica* | Well-watered | 25°C - 29°C | -0.011 | 0.00 | 0.114 |  |
| *g*_s_ | *F. sylvatica* | Well-watered | 29°C - 33°C | -0.004 | 0.00 | 0.879 |  |
| *g*_s_ | *F. sylvatica* | Well-watered | 33°C - 37°C | 0.006 | 0.00 | 0.435 |  |
| *g*_s_ | *F. sylvatica* | Well-watered | 37°C - 41°C | 0.010 | 0.00 | 0.038 |  |
| *g*_s_ | *F. sylvatica* | Well-watered | 41°C - 45°C | 0.008 | 0.00 | 0.534 |  |
| *g*_s_ | *F. sylvatica* | Drought | 25°C - 45°C | -0.003 | 0.01 | 0.996 |  |
| *g*_s_ | *F. sylvatica* | Drought | 25°C - 29°C | -0.003 | 0.00 | 0.862 |  |
| *g*_s_ | *F. sylvatica* | Drought | 29°C - 33°C | -0.002 | 0.00 | 0.889 |  |
| *g*_s_ | *F. sylvatica* | Drought | 33°C - 37°C | 0.000 | 0.00 | 1.000 |  |
| *g*_s_ | *F. sylvatica* | Drought | 37°C - 41°C | 0.001 | 0.00 | 0.994 |  |
| *g*_s_ | *F. sylvatica* | Drought | 41°C - 45°C | 0.002 | 0.00 | 0.988 |  |
| *g*_s_ | *Q. robur* | Well-watered | 25°C - 45°C | 0.017 | 0.01 | 0.141 |  |
| *g*_s_ | *Q. robur* | Well-watered | 25°C - 29°C | -0.012 | 0.00 | 0.120 |  |
| *g*_s_ | *Q. robur* | Well-watered | 29°C - 33°C | -0.008 | 0.00 | 0.338 |  |
| *g*_s_ | *Q. robur* | Well-watered | 33°C - 37°C | 0.004 | 0.00 | 0.920 |  |
| *g*_s_ | *Q. robur* | Well-watered | 37°C - 41°C | 0.015 | 0.00 | 0.004 |  |
| *g*_s_ | *Q. robur* | Well-watered | 41°C - 45°C | 0.018 | 0.01 | 0.019 |  |
| *g*_s_ | *Q. robur* | Drought | 25°C - 45°C | 0.008 | 0.01 | 0.726 |  |
| *g*_s_ | *Q. robur* | Drought | 25°C - 29°C | 0.002 | 0.00 | 0.726 |  |
| *g*_s_ | *Q. robur* | Drought | 29°C - 33°C | 0.002 | 0.00 | 0.726 |  |
| *g*_s_ | *Q. robur* | Drought | 33°C - 37°C | 0.002 | 0.00 | 0.726 |  |
| *g*_s_ | *Q. robur* | Drought | 37°C - 41°C | 0.002 | 0.00 | 0.726 |  |
| *g*_s_ | *Q. robur* | Drought | 41°C - 45°C | 0.002 | 0.00 | 0.726 |  |
| *E* | *A. platanoides* | Well-watered | 25°C - 45°C | -1.247 | 0.17 | < 0.001 | |
| *E* | *A. platanoides* | Well-watered | 25°C - 29°C | -0.249 | 0.03 | < 0.001 | |
| *E* | *A. platanoides* | Well-watered | 29°C - 33°C | -0.249 | 0.03 | < 0.001 | |
| *E* | *A. platanoides* | Well-watered | 33°C - 37°C | -0.249 | 0.03 | < 0.001 | |
| *E* | *A. platanoides* | Well-watered | 37°C - 41°C | -0.249 | 0.03 | < 0.001 | |
| *E* | *A. platanoides* | Well-watered | 41°C - 45°C | -0.249 | 0.03 | < 0.001 | |
| *E* | *A. platanoides* | Drought | 25°C - 45°C | -0.754 | 0.19 | 0.002 | |
| *E* | *A. platanoides* | Drought | 25°C - 29°C | -0.151 | 0.04 | 0.002 | |
| *E* | *A. platanoides* | Drought | 29°C - 33°C | -0.151 | 0.04 | 0.002 | |
| *E* | *A. platanoides* | Drought | 33°C - 37°C | -0.151 | 0.04 | 0.002 | |
| *E* | *A. platanoides* | Drought | 37°C - 41°C | -0.151 | 0.04 | 0.002 | |
| *E* | *A. platanoides* | Drought | 41°C - 45°C | -0.151 | 0.04 | 0.002 | |
| *E* | *F. sylvatica* | Well-watered | 25°C - 45°C | -1.688 | 0.21 | < 0.001 | |
| *E* | *F. sylvatica* | Well-watered | 25°C - 29°C | -0.338 | 0.04 | < 0.001 | |
| *E* | *F. sylvatica* | Well-watered | 29°C - 33°C | -0.338 | 0.04 | < 0.001 | |
| *E* | *F. sylvatica* | Well-watered | 33°C - 37°C | -0.337 | 0.04 | < 0.001 | |
| *E* | *F. sylvatica* | Well-watered | 37°C - 41°C | -0.337 | 0.04 | < 0.001 | |
| *E* | *F. sylvatica* | Well-watered | 41°C - 45°C | -0.337 | 0.04 | < 0.001 | |
| *E* | *F. sylvatica* | Drought | 25°C - 45°C | -1.161 | 0.22 | < 0.001 | |
| *E* | *F. sylvatica* | Drought | 25°C - 29°C | -0.232 | 0.04 | < 0.001 | |
| *E* | *F. sylvatica* | Drought | 29°C - 33°C | -0.232 | 0.04 | < 0.001 | |
| *E* | *F. sylvatica* | Drought | 33°C - 37°C | -0.232 | 0.04 | < 0.001 | |
| *E* | *F. sylvatica* | Drought | 37°C - 41°C | -0.232 | 0.04 | < 0.001 | |
| *E* | *F. sylvatica* | Drought | 41°C - 45°C | -0.232 | 0.04 | < 0.001 | |
| *E* | *Q. robur* | Well-watered | 25°C - 45°C | -1.725 | 0.28 | < 0.001 | |
| *E* | *Q. robur* | Well-watered | 25°C - 29°C | -0.609 | 0.17 | 0.010 | |
| *E* | *Q. robur* | Well-watered | 29°C - 33°C | -0.604 | 0.14 | < 0.001 | |
| *E* | *Q. robur* | Well-watered | 33°C - 37°C | -0.433 | 0.14 | 0.023 | |
| *E* | *Q. robur* | Well-watered | 37°C - 41°C | -0.145 | 0.13 | 0.889 | |
| *E* | *Q. robur* | Well-watered | 41°C - 45°C | 0.066 | 0.20 | 0.999 | |
| *E* | *Q. robur* | Drought | 25°C - 45°C | -1.359 | 0.26 | < 0.001 | |
| *E* | *Q. robur* | Drought | 25°C - 29°C | -0.272 | 0.05 | < 0.001 | |
| *E* | *Q. robur* | Drought | 29°C - 33°C | -0.272 | 0.05 | < 0.001 | |
| *E* | *Q. robur* | Drought | 33°C - 37°C | -0.272 | 0.05 | < 0.001 | |
| *E* | *Q. robur* | Drought | 37°C - 41°C | -0.272 | 0.05 | < 0.001 | |
| *E* | *Q. robur* | Drought | 41°C - 45°C | -0.272 | 0.05 | < 0.001 | |
| *WUE*_a_ | *A. platanoides* | Well-watered | 25°C - 45°C | 5.097 | 0.29 | < 0.001 | |
| *WUE*_a_ | *A. platanoides* | Well-watered | 25°C - 29°C | 1.464 | 0.19 | < 0.001 | |
| *WUE*_a_ | *A. platanoides* | Well-watered | 29°C - 33°C | 1.243 | 0.14 | < 0.001 | |
| *WUE*_a_ | *A. platanoides* | Well-watered | 33°C - 37°C | 0.918 | 0.14 | < 0.001 | |
| *WUE*_a_ | *A. platanoides* | Well-watered | 37°C - 41°C | 0.746 | 0.14 | < 0.001 | |
| *WUE*_a_ | *A. platanoides* | Well-watered | 41°C - 45°C | 0.726 | 0.21 | 0.014 | |
| *WUE*_a_ | *A. platanoides* | Drought | 25°C - 45°C | 5.502 | 0.30 | < 0.001 | |
| *WUE*_a_ | *A. platanoides* | Drought | 25°C - 29°C | 1.218 | 0.14 | < 0.001 | |
| *WUE*_a_ | *A. platanoides* | Drought | 29°C - 33°C | 1.157 | 0.10 | < 0.001 | |
| *WUE*_a_ | *A. platanoides* | Drought | 33°C - 37°C | 1.069 | 0.09 | < 0.001 | |
| *WUE*_a_ | *A. platanoides* | Drought | 37°C - 41°C | 1.026 | 0.10 | < 0.001 | |
| *WUE*_a_ | *A. platanoides* | Drought | 41°C - 45°C | 1.032 | 0.14 | < 0.001 | |
| *WUE*_a_ | *F. sylvatica* | Well-watered | 25°C - 45°C | 3.771 | 0.15 | < 0.001 | |
| *WUE*_a_ | *F. sylvatica* | Well-watered | 25°C - 29°C | 1.122 | 0.11 | < 0.001 | |
| *WUE*_a_ | *F. sylvatica* | Well-watered | 29°C - 33°C | 0.818 | 0.10 | < 0.001 | |
| *WUE*_a_ | *F. sylvatica* | Well-watered | 33°C - 37°C | 0.612 | 0.10 | < 0.001 | |
| *WUE*_a_ | *F. sylvatica* | Well-watered | 37°C - 41°C | 0.538 | 0.09 | < 0.001 | |
| *WUE*_a_ | *F. sylvatica* | Well-watered | 41°C - 45°C | 0.681 | 0.12 | < 0.001 | |
| *WUE*_a_ | *F. sylvatica* | Drought | 25°C - 45°C | 4.915 | 0.15 | < 0.001 | |
| *WUE*_a_ | *F. sylvatica* | Drought | 25°C - 29°C | 1.312 | 0.12 | < 0.001 | |
| *WUE*_a_ | *F. sylvatica* | Drought | 29°C - 33°C | 0.953 | 0.10 | < 0.001 | |
| *WUE*_a_ | *F. sylvatica* | Drought | 33°C - 37°C | 0.808 | 0.10 | < 0.001 | |
| *WUE*_a_ | *F. sylvatica* | Drought | 37°C - 41°C | 0.889 | 0.09 | < 0.001 | |
| *WUE*_a_ | *F. sylvatica* | Drought | 41°C - 45°C | 0.953 | 0.12 | < 0.001 | |
| *WUE*_a_ | *Q. robur* | Well-watered | 25°C - 45°C | 3.481 | 0.48 | < 0.001 | |
| *WUE*_a_ | *Q. robur* | Well-watered | 25°C - 29°C | 0.696 | 0.10 | < 0.001 | |
| *WUE*_a_ | *Q. robur* | Well-watered | 29°C - 33°C | 0.696 | 0.10 | < 0.001 | |
| *WUE*_a_ | *Q. robur* | Well-watered | 33°C - 37°C | 0.696 | 0.10 | < 0.001 | |
| *WUE*_a_ | *Q. robur* | Well-watered | 37°C - 41°C | 0.696 | 0.10 | < 0.001 | |
| *WUE*_a_ | *Q. robur* | Well-watered | 41°C - 45°C | 0.696 | 0.10 | < 0.001 | |
| *WUE*_a_ | *Q. robur* | Drought | 25°C - 45°C | 3.612 | 0.52 | < 0.001 | |
| *WUE*_a_ | *Q. robur* | Drought | 25°C - 29°C | 0.132 | 0.38 | 0.999 | |
| *WUE*_a_ | *Q. robur* | Drought | 29°C - 33°C | 0.790 | 0.34 | 0.189 | |
| *WUE*_a_ | *Q. robur* | Drought | 33°C - 37°C | 1.469 | 0.33 | < 0.001 | |
| *WUE*_a_ | *Q. robur* | Drought | 37°C - 41°C | 0.919 | 0.32 | 0.056 | |
| *WUE*_a_ | *Q. robur* | Drought | 41°C - 45°C | 0.302 | 0.41 | 0.978 | |
| *WUE*_i_ | *A. platanoides* | Well-watered | 25°C - 45°C | 59.826 | 13.11 | < 0.001 | |
| *WUE*_i_ | *A. platanoides* | Well-watered | 25°C - 29°C | 11.963 | 2.63 | < 0.001 | |
| *WUE*_i_ | *A. platanoides* | Well-watered | 29°C - 33°C | 11.964 | 2.62 | < 0.001 | |
| *WUE*_i_ | *A. platanoides* | Well-watered | 33°C - 37°C | 11.965 | 2.62 | < 0.001 | |
| *WUE*_i_ | *A. platanoides* | Well-watered | 37°C - 41°C | 11.967 | 2.62 | < 0.001 | |
| *WUE*_i_ | *A. platanoides* | Well-watered | 41°C - 45°C | 11.968 | 2.63 | < 0.001 | |
| *WUE*_i_ | *A. platanoides* | Drought | 25°C - 45°C | 193.692 | 16.36 | < 0.001 | |
| *WUE*_i_ | *A. platanoides* | Drought | 25°C - 29°C | 16.324 | 13.41 | 0.827 | |
| *WUE*_i_ | *A. platanoides* | Drought | 29°C - 33°C | 18.581 | 10.21 | 0.460 | |
| *WUE*_i_ | *A. platanoides* | Drought | 33°C - 37°C | 21.885 | 10.50 | 0.308 | |
| *WUE*_i_ | *A. platanoides* | Drought | 37°C - 41°C | 46.829 | 10.33 | < 0.001 | |
| *WUE*_i_ | *A. platanoides* | Drought | 41°C - 45°C | 90.072 | 13.29 | < 0.001 | |
| *WUE*_i_ | *F. sylvatica* | Well-watered | 25°C - 45°C | 47.619 | 7.34 | < 0.001 | |
| *WUE*_i_ | *F. sylvatica* | Well-watered | 25°C - 29°C | 11.306 | 5.69 | 0.357 | |
| *WUE*_i_ | *F. sylvatica* | Well-watered | 29°C - 33°C | 5.148 | 5.20 | 0.920 | |
| *WUE*_i_ | *F. sylvatica* | Well-watered | 33°C - 37°C | -0.004 | 5.15 | 1.000 | |
| *WUE*_i_ | *F. sylvatica* | Well-watered | 37°C - 41°C | 4.919 | 4.90 | 0.916 | |
| *WUE*_i_ | *F. sylvatica* | Well-watered | 41°C - 45°C | 26.250 | 6.26 | < 0.001 | |
| *WUE*_i_ | *F. sylvatica* | Drought | 25°C - 45°C | 124.189 | 7.82 | < 0.001 | |
| *WUE*_i_ | *F. sylvatica* | Drought | 25°C - 29°C | 16.207 | 6.65 | 0.153 | |
| *WUE*_i_ | *F. sylvatica* | Drought | 29°C - 33°C | 7.996 | 6.27 | 0.798 | |
| *WUE*_i_ | *F. sylvatica* | Drought | 33°C - 37°C | 10.146 | 6.10 | 0.558 | |
| *WUE*_i_ | *F. sylvatica* | Drought | 37°C - 41°C | 31.747 | 5.70 | < 0.001 | |
| *WUE*_i_ | *F. sylvatica* | Drought | 41°C - 45°C | 58.094 | 6.78 | < 0.001 | |
| *WUE*_i_ | *Q. robur* | Well-watered | 25°C - 45°C | 9.717 | 18.13 | 0.995 | |
| *WUE*_i_ | *Q. robur* | Well-watered | 25°C - 29°C | 1.915 | 3.67 | 0.995 | |
| *WUE*_i_ | *Q. robur* | Well-watered | 29°C - 33°C | 1.927 | 3.64 | 0.995 | |
| *WUE*_i_ | *Q. robur* | Well-watered | 33°C - 37°C | 1.941 | 3.63 | 0.995 | |
| *WUE*_i_ | *Q. robur* | Well-watered | 37°C - 41°C | 1.958 | 3.64 | 0.994 | |
| *WUE*_i_ | *Q. robur* | Well-watered | 41°C - 45°C | 1.975 | 3.67 | 0.994 | |
| *WUE*_i_ | *Q. robur* | Drought | 25°C - 45°C | 82.028 | 19.97 | 0.001 | |
| *WUE*_i_ | *Q. robur* | Drought | 25°C - 29°C | -33.547 | 15.79 | 0.286 | |
| *WUE*_i_ | *Q. robur* | Drought | 29°C - 33°C | 0.487 | 16.65 | 1.000 | |
| *WUE*_i_ | *Q. robur* | Drought | 33°C - 37°C | 67.639 | 15.57 | < 0.001 | |
| *WUE*_i_ | *Q. robur* | Drought | 37°C - 41°C | 39.886 | 15.37 | 0.111 | |
| *WUE*_i_ | *Q. robur* | Drought | 41°C - 45°C | 7.563 | 17.78 | 0.998 | |

**Table S6** Parametric coefficients from species-specific generalized linear mixed models (glmmTMB), assessing the effect of T_air_, drought treatment and their interaction on T_leaf_.

|  | ***A. platanoides*** | ***F. sylvatica*** | ***Q. robur*** |
| --- | --- | --- | --- |
| (Intercept) | 44.248 *** | 42.136 *** | 41.923 *** |
|  | (0.772) | (0.463) | (0.573) |
| Drought | 2.990 ** | 2.892 *** | 5.652 *** |
|  | (1.029) | (0.675) | (0.809) |
| Tair | 0.774 *** | 0.815 *** | 0.813 *** |
|  | (0.047) | (0.027) | (0.027) |
| Drought:Tair | 0.169 ** | 0.109 ** | 0.256 *** |
|  | (0.056) | (0.039) | (0.038) |
| N | 76 | 113 | 84 |
| logLik | -155.683 | -214.698 | -150.467 |
| AIC | 325.366 | 441.396 | 312.933 |

*** p < 0.001; ** p < 0.01; * p < 0.05.

**Table S7** Parametric coefficients from species-specific generalized linear mixed models (glmmTMB), assessing the effect of E, drought treatment and their interaction on T_diff_.

|  | ***A. platanoides*** | ***F. sylvatica*** | ***Q. robur*** |
| --- | --- | --- | --- |
| (Intercept) | 2.975 ** | 1.308 | 3.202 ** |
|  | (1.132) | (0.740) | (1.014) |
| E | -1.256 * | -1.364 *** | -1.876 *** |
|  | (0.583) | (0.296) | (0.354) |
| Drought | 1.276 | 0.176 | -2.209 |
|  | (1.661) | (0.979) | (1.332) |
| E:Drought | -0.347 | 0.591 | 2.384 *** |
|  | (1.190) | (0.486) | (0.515) |
| N | 64 | 93 | 84 |
| logLik | -137.097 | -177.203 | -157.758 |
| AIC | 286.194 | 366.406 | 327.516 |
| *** p < 0.001; ** p < 0.01; * p < 0.05. | | | |

**Table S8** Parametric coefficients from species-specific linear models, assessing the effect of drought treatment on the amount of leaf damage (%) at the end of the experiment. No model was fitted to *Q. robur,* as no leaf damage was observed.

|  | ***A. platanoides*** | ***F. sylvatica*** |
| --- | --- | --- |
| Intercept | 11.840 * | 9.865 * |
|  | (4.658) | (4.374) |
| Drought | 39.940 *** | 15.228 * |
|  | (6.857) | (6.355) |
| N | 13 | 19 |
| R2 | 0.755 | 0.252 |
| logLik | -50.012 | -75.816 |
| AIC | 106.024 | 157.632 |

*** p < 0.001; ** p < 0.01; * p < 0.05.

**Table S9** Parametric coefficients from species-specific generalized linear mixed models (glmmTMB) evaluating the effect of different time steps (pre = at 25°C, 45°C = final day of heat stress, R1 = one day post heat stress, R 2 = two days post heat stress) and drought treatment on gas-exchange responses.

|  | ***A. platanoides*** | | ***F. sylvatica*** | | ***Q. robur*** | |
| --- | --- | --- | --- | --- | --- | --- |
|  | **A_net_** | **gₛ** | **A_net_** | **gₛ** | **A_net_** | **gₛ** |
| (Intercept) | 4.843 *** | 0.047 *** | 4.703 *** | 0.045 *** | 5.391 *** | 0.051 *** |
|  | (0.278) | (0.003) | (0.336) | (0.004) | (0.395) | (0.005) |
| R1 | -2.635 *** | -0.015 *** | -1.614 *** | -0.010 *** | 1.318 ** | 0.012 *** |
|  | (0.138) | (0.002) | (0.164) | (0.002) | (0.441) | (0.003) |
| R2 | -2.583 *** | -0.015 *** | -1.392 *** | -0.010 *** | 1.349 ** | 0.025 *** |
|  | (0.138) | (0.002) | (0.181) | (0.002) | (0.445) | (0.003) |
| 45°C | -3.396 *** | -0.016 *** | -2.811 *** | -0.006 ** | -1.836 *** | -0.014 *** |
|  | (0.112) | (0.001) | (0.137) | (0.002) | (0.437) | (0.003) |
| Drought | -2.394 *** | -0.030 *** | -2.025 *** | -0.028 *** | -1.097 * | -0.017 ** |
|  | (0.393) | (0.005) | (0.487) | (0.005) | (0.557) | (0.006) |
| R1:Drought | 0.454 * | 0.011 *** | 0.193 | 0.009 ** | -2.849 *** | -0.022 *** |
|  | (0.195) | (0.002) | (0.236) | (0.003) | (0.623) | (0.004) |
| R2:Drought | 0.505 ** | 0.013 *** | 0.204 | 0.006 * | -2.165 *** | -0.028 *** |
|  | (0.195) | (0.002) | (0.254) | (0.003) | (0.623) | (0.004) |
| 45°C:Drought | 0.307 | 0.012 *** | 0.373 | 0.010 *** | -1.959 ** | 0.005 |
|  | (0.158) | (0.002) | (0.198) | (0.002) | (0.617) | (0.004) |
| N | 456 | 455 | 468 | 468 | 441 | 440 |
| logLik | -502.184 | 1554.066 | -622.459 | 1429.415 | -1012.018 | 1200.763 |
| AIC | 1024.367 | -3088.131 | 1264.917 | -2838.829 | 2044.035 | -2381.526 |

**Table S10** Estimated marginal means and post-hoc Tukey contrasts at different time steps (pre = at 25°C, 45°C = final day of heat stress, R1 = one day post heat stress, R 2 = two days post heat stress) per watering treatment, gas-exchange variable and species. Results are obtained from generalized linear mixed models (glmmTMB) fitted independently for each species and gas-exchange variable.

| Variable | Species | Treatment | Comparison | Estimate | Std. Error | p-value |
| --- | --- | --- | --- | --- | --- | --- |
| Aₙₑₜ | *A. platanoides* | Well-watered | pre - R1 | 2.635 | 0.14 | < 0.001 |
| Aₙₑₜ | *A. platanoides* | Well-watered | pre - R2 | 2.583 | 0.14 | < 0.001 |
| Aₙₑₜ | *A. platanoides* | Well-watered | pre - stress | 3.396 | 0.11 | < 0.001 |
| Aₙₑₜ | *A. platanoides* | Well-watered | R1 - R2 | -0.052 | 0.16 | 0.989 |
| Aₙₑₜ | *A. platanoides* | Well-watered | R1 - stress | 0.761 | 0.14 | < 0.001 |
| Aₙₑₜ | *A. platanoides* | Well-watered | R2 - stress | 0.813 | 0.14 | < 0.001 |
| Aₙₑₜ | *A. platanoides* | Drought | pre - R1 | 2.181 | 0.14 | < 0.001 |
| Aₙₑₜ | *A. platanoides* | Drought | pre - R2 | 2.078 | 0.14 | < 0.001 |
| Aₙₑₜ | *A. platanoides* | Drought | pre - stress | 3.089 | 0.11 | < 0.001 |
| Aₙₑₜ | *A. platanoides* | Drought | R1 - R2 | -0.103 | 0.16 | 0.923 |
| Aₙₑₜ | *A. platanoides* | Drought | R1 - stress | 0.908 | 0.14 | < 0.001 |
| Aₙₑₜ | *A. platanoides* | Drought | R2 - stress | 1.011 | 0.14 | < 0.001 |
| Aₙₑₜ | *F. sylvatica* | Well-watered | pre - R1 | 1.614 | 0.16 | < 0.001 |
| Aₙₑₜ | *F. sylvatica* | Well-watered | pre - R2 | 1.392 | 0.18 | < 0.001 |
| Aₙₑₜ | *F. sylvatica* | Well-watered | pre - stress | 2.811 | 0.14 | < 0.001 |
| Aₙₑₜ | *F. sylvatica* | Well-watered | R1 - R2 | -0.222 | 0.19 | 0.657 |
| Aₙₑₜ | *F. sylvatica* | Well-watered | R1 - stress | 1.197 | 0.15 | < 0.001 |
| Aₙₑₜ | *F. sylvatica* | Well-watered | R2 - stress | 1.419 | 0.17 | < 0.001 |
| Aₙₑₜ | *F. sylvatica* | Drought | pre - R1 | 1.421 | 0.17 | < 0.001 |
| Aₙₑₜ | *F. sylvatica* | Drought | pre - R2 | 1.189 | 0.18 | < 0.001 |
| Aₙₑₜ | *F. sylvatica* | Drought | pre - stress | 2.438 | 0.14 | < 0.001 |
| Aₙₑₜ | *F. sylvatica* | Drought | R1 - R2 | -0.232 | 0.19 | 0.623 |
| Aₙₑₜ | *F. sylvatica* | Drought | R1 - stress | 1.018 | 0.16 | < 0.001 |
| Aₙₑₜ | *F. sylvatica* | Drought | R2 - stress | 1.249 | 0.17 | < 0.001 |
| Aₙₑₜ | *Q. robur* | Well-watered | pre - R1 | -1.318 | 0.44 | 0.015 |
| Aₙₑₜ | *Q. robur* | Well-watered | pre - R2 | -1.349 | 0.45 | 0.013 |
| Aₙₑₜ | *Q. robur* | Well-watered | pre - stress | 1.836 | 0.44 | < 0.001 |
| Aₙₑₜ | *Q. robur* | Well-watered | R1 - R2 | -0.031 | 0.53 | 1.000 |
| Aₙₑₜ | *Q. robur* | Well-watered | R1 - stress | 3.154 | 0.53 | < 0.001 |
| Aₙₑₜ | *Q. robur* | Well-watered | R2 - stress | 3.185 | 0.53 | < 0.001 |
| Aₙₑₜ | *Q. robur* | Drought | pre - R1 | 1.530 | 0.44 | 0.003 |
| Aₙₑₜ | *Q. robur* | Drought | pre - R2 | 0.816 | 0.44 | 0.239 |
| Aₙₑₜ | *Q. robur* | Drought | pre - stress | 3.795 | 0.44 | < 0.001 |
| Aₙₑₜ | *Q. robur* | Drought | R1 - R2 | -0.714 | 0.53 | 0.527 |
| Aₙₑₜ | *Q. robur* | Drought | R1 - stress | 2.265 | 0.53 | < 0.001 |
| Aₙₑₜ | *Q. robur* | Drought | R2 - stress | 2.979 | 0.52 | < 0.001 |
| gₛ | *A. platanoides* | Well-watered | pre - R1 | 0.015 | 0 | < 0.001 |
| gₛ | *A. platanoides* | Well-watered | pre - R2 | 0.015 | 0 | < 0.001 |
| gₛ | *A. platanoides* | Well-watered | pre - stress | 0.016 | 0 | < 0.001 |
| gₛ | *A. platanoides* | Well-watered | R1 - R2 | 0.001 | 0 | 0.967 |
| gₛ | *A. platanoides* | Well-watered | R1 - stress | 0.001 | 0 | 0.806 |
| gₛ | *A. platanoides* | Well-watered | R2 - stress | 0.001 | 0 | 0.984 |
| gₛ | *A. platanoides* | Drought | pre - R1 | 0.003 | 0 | 0.147 |
| gₛ | *A. platanoides* | Drought | pre - R2 | 0.002 | 0 | 0.447 |
| gₛ | *A. platanoides* | Drought | pre - stress | 0.004 | 0 | 0.013 |
| gₛ | *A. platanoides* | Drought | R1 - R2 | -0.001 | 0 | 0.951 |
| gₛ | *A. platanoides* | Drought | R1 - stress | 0.000 | 0 | 0.989 |
| gₛ | *A. platanoides* | Drought | R2 - stress | 0.001 | 0 | 0.787 |
| gₛ | *F. sylvatica* | Well-watered | pre - R1 | 0.010 | 0 | < 0.001 |
| gₛ | *F. sylvatica* | Well-watered | pre - R2 | 0.010 | 0 | < 0.001 |
| gₛ | *F. sylvatica* | Well-watered | pre - stress | 0.006 | 0 | 0.008 |
| gₛ | *F. sylvatica* | Well-watered | R1 - R2 | -0.001 | 0 | 0.992 |
| gₛ | *F. sylvatica* | Well-watered | R1 - stress | -0.005 | 0 | 0.058 |
| gₛ | *F. sylvatica* | Well-watered | R2 - stress | -0.004 | 0 | 0.219 |
| gₛ | *F. sylvatica* | Drought | pre - R1 | 0.002 | 0 | 0.893 |
| gₛ | *F. sylvatica* | Drought | pre - R2 | 0.003 | 0 | 0.477 |
| gₛ | *F. sylvatica* | Drought | pre - stress | -0.004 | 0 | 0.060 |
| gₛ | *F. sylvatica* | Drought | R1 - R2 | 0.002 | 0 | 0.899 |
| gₛ | *F. sylvatica* | Drought | R1 - stress | -0.006 | 0 | 0.016 |
| gₛ | *F. sylvatica* | Drought | R2 - stress | -0.008 | 0 | 0.002 |
| gₛ | *Q. robur* | Well-watered | pre - R1 | -0.012 | 0 | < 0.001 |
| gₛ | *Q. robur* | Well-watered | pre - R2 | -0.025 | 0 | < 0.001 |
| gₛ | *Q. robur* | Well-watered | pre - stress | 0.014 | 0 | < 0.001 |
| gₛ | *Q. robur* | Well-watered | R1 - R2 | -0.013 | 0 | 0.001 |
| gₛ | *Q. robur* | Well-watered | R1 - stress | 0.026 | 0 | < 0.001 |
| gₛ | *Q. robur* | Well-watered | R2 - stress | 0.039 | 0 | < 0.001 |
| gₛ | *Q. robur* | Drought | pre - R1 | 0.010 | 0 | 0.003 |
| gₛ | *Q. robur* | Drought | pre - R2 | 0.003 | 0 | 0.670 |
| gₛ | *Q. robur* | Drought | pre - stress | 0.009 | 0 | 0.011 |
| gₛ | *Q. robur* | Drought | R1 - R2 | -0.007 | 0 | 0.210 |
| gₛ | *Q. robur* | Drought | R1 - stress | -0.001 | 0 | 0.986 |
| gₛ | *Q. robur* | Drought | R2 - stress | 0.005 | 0 | 0.371 |
